# Efficacy of Various Virtual Reality Exposure Therapies for Chronic Low Back Pain: Systematic Review and Network Meta-Analysis

**DOI:** 10.2196/90289

**Published:** 2026-07-22

**Authors:** Linjie Wu, Xingyu Liu, Qichao Yin, Tianqi Yao, Yanhu Li

**Affiliations:** 1 Beijing Sport University, Beijing, China; 2 College of Mathematics and Systems Science, Shandong University of Science and Technology, Qingdao, China; 3 National Institute of Sports Medicine, General Administration of Sport of China, No 2 Jia, 3rd Floor, Tiyuguan Road, Dongcheng District, Beijing, 100061, China, 86 18813095716

**Keywords:** virtual reality, virtual reality–based training, chronic, low back pain, rehabilitation, PRISMA, Preferred Reporting Items for Systematic Reviews and Meta-Analyses

## Abstract

**Background:**

Chronic low back pain (CLBP) is a major global health challenge. While nonpharmacological therapies are recommended, patient compliance is often hindered by kinesiophobia. Virtual reality (VR) offers an immersive, distraction-based approach, but the comparative effectiveness of different VR modalities remains unclear.

**Objective:**

The aim of the study is to compare and rank the efficacy of different VR-based training modalities on pain intensity, disability, and kinesiophobia in patients with CLBP.

**Methods:**

Systematic searches were conducted in PubMed, Web of Science, Scopus, Embase, CINAHL, and the Cochrane Library from inception until June 2025. Randomized controlled trials (RCTs) assessing the effects of VR-based training on individuals with CLBP were selected. Primary outcomes were pain intensity, disability (Oswestry Disability Index), and kinesiophobia (Tampa Scale of Kinesiophobia). The Cochrane Risk of Bias 2 tool was used for quality assessment. Confidence in Network Meta-Analysis (CINeMA) framework was used to evaluate the credibility of cumulative evidence. A Bayesian network meta-analysis with standardized mean difference (SMD) as effect size was performed to synthesize evidence and rank interventions using surface under the cumulative ranking curve values. The GRADE (Grading of Recommendations Assessment, Development and Evaluation) framework was adapted to evaluate the quality of evidence.

**Results:**

In total, 25 RCTs with a total of 2610 participants were included in the analysis. For pain intensity, shooting games (SMD −4.40, 95% credible interval [CrI] −6.80 to −2.20) and VR-based equestrian training (SMD −2.00, 95% CrI −3.70 to −0.57) were significantly superior to all types of controls. Surface under the cumulative ranking curve indicated that shooting games had the highest probability (98%) of being the most effective intervention for pain relief. For disability, no intervention demonstrated statistically significant superiority. For kinesiophobia, shooting games (SMD −3.40, 95% CrI −5.60 to −1.10) significantly outperformed traditional exercise controls. The quality of evidence ranged from very low to moderate across outcomes.

**Conclusions:**

This first network meta-analysis to compare and rank distinct VR modalities for CLBP offers several key innovations and contributions to the field. By moving beyond aggregate VR categorizations, we provide a granular, comparative ranking of specific, actionable VR interventions. Unlike previous reviews that treated VR as a homogeneous group or only compared it to sham, our network meta-analysis directly and indirectly compares 7 distinct VR modalities, revealing that not all VR is equally effective. Our findings suggest that shooting games have the potential to be the most effective VR therapy for relieving pain intensity and kinesiophobia, though evidence for disability remains limited. Unfortunately, due to heterogeneity and low-quality evidence, there is no evidence demonstrating significant improvement in specific outcomes for patients with CLBP. More RCTs are needed to provide robust clinical evidence.

## Introduction

Chronic low back pain (CLBP) is defined as a pain syndrome lasting at least 12 weeks, localized below the lower costal margin, above the gluteal fold, and between the bilateral midaxillary lines [1,2]. Epidemiological evidence indicates that approximately 84% of individuals experience low back pain during their lifetime, with CLBP accounting for 23% of these cases and leading to disability in 11%‐12% of the global population [3,4], thus representing a major global health challenge [5]. Although nonpharmacological interventions (including exercise therapy, cognitive behavioral therapy, and manual therapy) are recommended as first-line treatments for CLBP in multiple clinical guidelines [6,7], their effectiveness is frequently limited by poor patient adherence and the presence of kinesiophobia [8,9]. Kinesiophobia, defined as an excessive fear of movement, triggers avoidance behaviors and perpetuates functional disability, thereby impeding the rehabilitation process [10]. To overcome this barrier, virtual reality (VR)–based training has emerged as a promising adjunctive rehabilitation approach. Accumulating evidence confirms that VR can effectively reduce kinesiophobia and improve treatment adherence in patients with low back pain [11-13].

VR is a computer-generated technology that enables 3D spatial interaction and immersive experience [14,15]. In clinical practice, VR exerts analgesic effects through immersive attentional distraction and graded exposure to feared movements [16,17]. VR modalities vary considerably in their content and interaction requirements. It can be broadly divided into active VR (involving physical interaction within virtual environments) and passive VR (serving primarily as a distraction tool) [18,19]. Notably, existing studies have preliminarily explored the therapeutic differences among various VR games; however, most studies classify VR interventions based on hardware characteristics, such as immersive, semi-immersive, and nonimmersive, rather than differentiating intervention effects according to physical engagement levels and specific therapeutic targets [11,18,20-25]. Consequently, the comparative efficacy of distinct VR content modalities for CLBP remains unclear.

Several preliminary studies have attempted to address this gap. For instance, Brea-Gómez et al [11] conducted subgroup analyses stratified by VR type and reported that equine simulator training significantly improved pain intensity, providing preliminary evidence for the differential effects of VR modalities. Li et al [22] suggested that future research on VR-based training should focus on intervention types, parameters, and demographic characteristics. Nevertheless, no network meta-analysis has systematically compared and ranked multiple distinct VR modalities for CLBP.

To address the lack of comparative evidence, this study aimed to perform a systematic review and network meta-analysis of randomized controlled trials (RCTs). The primary objective was to evaluate the comparative efficacy of different VR interventions on outcomes of pain intensity, disability, and kinesiophobia, with the goal of identifying the most effective approaches for clinical practice.

## Methods

### Study Design

The PRISMA (Preferred Reporting Items for Systematic Reviews and Meta-Analyses) 2020 declaration was followed in this systematic review and was checked against the PRISMA 2020 expanded checklist in Checklist 1 [26]. The study protocol was prospectively recorded in the International Prospective Register of Systematic Reviews (PROSPERO: 2025 CRD420251131116).

### Search Strategy

This study adhered to the PRISMA-S (Preferred Reporting Items for Systematic Reviews and Meta-Analyses Literature Search Extension) checklist (Checklist 2) [26] extension to systematically document the literature search strategy and process. Information sources included the following databases and their respective platforms: PubMed (via NCBI), Cochrane Library (via Wiley), Web of Science Core Collection (via Clarivate), Embase (via Ovid), CINAHL (via EBSCOhost), and Scopus (via Elsevier). Backward and forward citation tracking was performed using reference lists and the citation index in Web of Science. Furthermore, we have traced and screened the studies included in relevant previous reviews to supplement the present review. Experts and corresponding authors were contacted by email to identify unpublished or ongoing studies. No additional sources or search methods were used.

Detailed search strategies combining subject headings and free-text terms were developed for each database; the full strategies are provided in Multimedia Appendix 1. Searches were limited to records from database inception to January 27, 2026, and to English-language publications. Published search filters for RCTs were adapted where applicable. The search approach was informed and updated from previously validated strategies in related reviews [22]. All strategies underwent peer review by an experienced information specialist and were refined accordingly. Literature collection and screening were conducted through EndNote (version 20; Clarivate).

### Eligibility Criteria

Inclusion and exclusion criteria were formulated according to the population, intervention, comparison, outcomes, and study design principles [27]：

- All of the included studies were RCTs.
- Sample requirements: Individuals with low back pain that persisted for more than 3 months.
- Intervention measures: VR games or VR-based interventions were used. This study included all types of VR interventions, such as fully immersive, semi-immersive, and nonimmersive, in order to maximize network construction. The control group was uniform if physical treatment was applied; the specifications of the VR device were not required.
- Non-VR–based therapies, including conventional exercise controls, nonexercise controls, or placebo controls, were administered to control groups.
- Outcome measures: The Department of Defense and Veterans Pain Rating Scale, visual analog scales, or Numerical Rating Scales, which have a 0‐10 point scoring system, were used to quantify the level of pain. The Tampa Scale of Kinesiophobia was used to measure fear associated with movement. The Oswestry Disability Index was used to measure disability. Textbox S1 in Multimedia Appendix 2 contained specific inclusion and exclusion criteria. This study used group-based processing since VR interventions and control measures varied greatly.

### Data Extraction and Selection Criteria

Double-blind screening was carried out independently by 2 reviewers (LW and XL) who first selected papers by looking at titles, abstracts, and keywords. Full-text screening against inclusion or exclusion criteria was performed on papers that satisfied the first criterion. To guarantee thorough literature coverage, secondary screening required obtaining reverse references. A third reviewer was consulted to address disagreements.

Two independent researchers extracted pertinent publication data (eg, authors, title, and year), study design (eg, RCTs and number of assessment time points), patient numbers, patient characteristics (eg, age, sex, and duration of pain), intervention measures, available outcome measures, and results. For all pre- and postintervention measures, outcome data were displayed as mean (SD). We translated data that were presented as discrete measures, such as the median, to mean (SD) [28].

### Risk of Bias

Based on quality evaluation criteria, the Cochrane Library’s Risk of Bias 2 tool was used to evaluate the quality of the included studies, primarily assessing RCTs across 5 domains: bias arising from the randomization process, bias due to deviations from intended interventions, bias due to missing outcome data, bias in measurement of the outcome, and bias in selection of the reported result [29]. The κ coefficient was used to measure methodological quality and risk of bias assessment consistency between the 2 independent reviewers. Any item-level disagreements arising during this process were resolved by arbitration from a third reviewer.

### Qualitative Analysis

The credibility of the cumulative evidence was evaluated using the Confidence in Network Meta-Analysis (CINeMA) framework [30,31] to categorize the evidence for each intervention across outcome measures into 4 levels: high, moderate, low, and very low [32,33]. Two independent reviewers (LW and XL) evaluated each study. Discrepancies were resolved through discussion, and a third reviewer (TY) adjudicated any unresolved disagreements.

### Statistical Analysis

A Bayesian network meta-analysis was performed using R software (v4.5.1; R Foundation for Statistical Computing) and RStudio (Posit Software, PBC) within a Markov Chain Monte Carlo framework [34]. Network diagrams illustrated direct comparisons between VR therapies, and heterogeneity was assessed using chi-square tests [35]. To maintain alignment with other indicators, a random-effects model was always used, regardless of whether significant heterogeneity was found. The standardized mean difference (SMD) and 95% credible intervals (CrIs) were used to report pooled effect sizes and precision. Results were presented in forest plots and effect estimate tables. Global consistency was evaluated by comparing deviance bar and deviance information criterion between consistent and inconsistent models, with a deviance information criterion difference <5 favoring consistency [36].

Model convergence was assessed via diagnostic plots (Figure S6, Multimedia Appendix 2); studies with shrinkage factors >1.02 were excluded via sensitivity analysis [37]. To explore heterogeneity sources, prespecified covariates—including types of VR interventions, intervention complexity, patient age, intervention duration, region, sample size, study year, and control type—were incorporated using Bayesian network meta-regression. A covariate was considered significant if the 95% CI of its regression coefficient excluded 0 [38]. Node-splitting analysis evaluated local inconsistency by testing differences between direct and indirect evidence. Treatments were ranked using surface under the cumulative ranking curve (SUCRA) and average rankings, with higher SUCRA values indicating better efficacy [39]. Publication bias was assessed using adjusted funnel plots and the Egger test (for outcomes with ≥10 studies); *P*<.05 and funnel plot asymmetry indicated potential bias, with the trim-and-fill method applied if small-study effects were detected [40].

## Results

### Results of Study Selection

**Figure 1.**
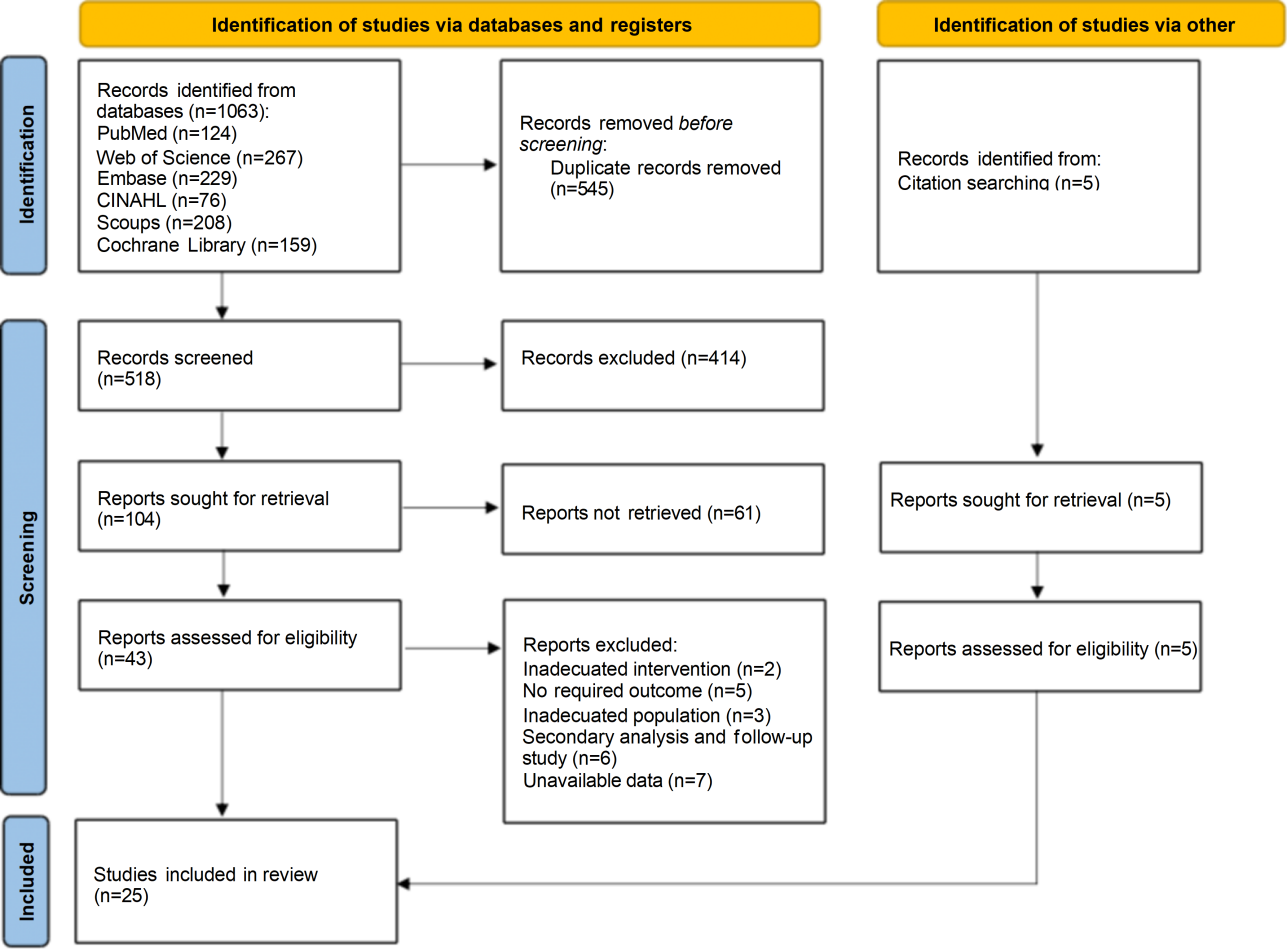
PRISMA (Preferred Reporting Items for Systematic Reviews and Meta-Analyses) flowchart of study selection.

The initial yield from each database was: PubMed (n=124), Embase (n=229), Cochrane Library (n=159), Web of Science (n=267), Scopus (n=208), CINHAL (n=76), and other sources (n=5). The initial search identified 1063 records from databases and 5 from other sources. After removing duplicates, 545 records were screened by title and abstract. Of these, 414 were excluded for being abstracts only, involving animal studies, conference proceedings, or not being RCTs. Full texts of the remaining 104 papers were assessed. Subsequently, 61 papers were excluded due to unavailability of full texts or trial registrations, and a further 23 were excluded for reasons including unsuitable interventions, missing relevant outcomes or participants, being secondary or follow-up analyses, or unavailable data. Finally, 25 studies [41-65] were included in the network meta-analysis (Figure 1).

### Study Characteristics

The 25 RCTs included in this study involved a total of 2183 patients with CLBP. The sample size of individual studies ranged from 18 to 1067 participants, with a mean age between 20 and 68 years and a generally balanced sex distribution. The interventions were categorized into 3 technical types: fully immersive VR (using head-mounted displays for complete visual immersion, 7 studies), semi-immersive systems (eg, large-screen projection combined with partial body interaction, 1 study), and nonimmersive desktop-based VR (monitor-based, 17 studies). Table 1 lists the author-assigned group names and definitions that were used to define the specific intervention protocols and control groups. In terms of dose parameters, the intervention duration varied from a single acute session to long-term programs lasting up to 12 weeks. The treatment frequency ranged from 2 to 5 sessions per week, with each session lasting 15 to 60 minutes, yielding a cumulative intervention time of 30 minutes to 36 hours. These dose parameters will be used for subsequent heterogeneity analysis and subgroup comparisons. Details are shown in Table 2.

**Table 1.** Definitions of interventions and controls in the text.

Type	Full name	Definition
Intervention		
VRBR^a^	VR^b^-based cognitive behavioral therapy	Based on cognitive behavioral therapy, the immersive VR experience incorporates pain education, mindfulness practice, interoceptive regulation, attention control, cognitive reappraisal, diaphragmatic breathing, graded exposure therapy, and additional techniques.
VRBO^c^	VR-based walking training	Based on aerobic exercise, the VR experience involves the completion of VR-based walking training through Vita Digital Productions.
VRBHm^d^	VR-based equestrian training	VR training with an equestrian simulator.
VRBHj^e^	Shooting games	VR training based on a shooting game.
VRBMix^f^	Multicomponent mixed games	Using a VR platform, this intervention is designed to promote full-body physical activity. It uses either a combination of sporting game modalities (eg, boxing, football, skiing, and rock climbing) or a structured program encompassing an amalgamation of 2 or more exercise components, including but not limited to aerobic, core strengthening, resistance, balance, coordination, and cognitive training.
VRBL^g^	Pelvic flexibility games	Using immersive or nonimmersive VR gaming to complete tasks, where pelvic tilt motions are used as the input to control virtual targets (such as caterpillars or fish) for rehabilitation or training purposes.
VRBY^h^	VR-based yoga training	A VR yoga training system that uses the Wii-Fit Balance Board as a platform for performing 7 specific asanas (eg, Half Moon, Warrior, and Tree Pose), enhancing immersion and biofeedback.
Control		
CON1^i^	Traditional exercise controls	The control group received conventional exercise therapy without VR, including core muscle training under exercise prescription, lumbar flexibility training, cognitive behavioral therapy, strength training, resistance training, and balance training.
CON2^j^	Nonexercise controls	The control group received sham VR, psychological interventions, pharmacotherapy, or physiotherapy (eg, ultrasound, heat, and magnetic therapy).
CON3^k^	Placebo controls	The control group received standard care without any intervention.

a VRBR: VR-based cognitive behavioral therapy.

b VR: virtual reality.

c VRBO: VR-based walking training.

d VRBHm: VR-based equestrian training.

e VRBHj: shooting game.

f VRBMix: multicomponent mixed game.

g VRBL: pelvic flexibility game.

h VRBY: VR-based yoga training.

i CON1: traditional exercise control.

j CON2: nonexercise control.

k CON3: placebo control.

**Table 2.** Characteristics and publication data of included studies.

Study (year)	Country	Type of work	Sample size	Sex	Mean age (years)	Pain duration (months)	Intervention	Exposure	Outcome measures	Follow-up points
Čeko et al (2024) [42]	United States	RCT^a^	EG^b^: 30 CG^c^: 30	Male: 31 Female: 30	34.3	≥3	EG: Psychotherapy includes pain education, mindfulness practice, interoceptive regulation, attention control, cognitive appraisal, diaphragmatic breathing, graded exposure therapy, and emotion regulation. CG: Usual care or waitlist.	14‐27 minutes per day 40 days	ODI^d^	2 weeks
Afzal et al (2022) [41]	Pakistan	RCT	EG: 42 CG: 42	Male: 28 Female: 56	EG: 38.2 CG: 37.5	—^f^	EG: VR^e^-based exergames+physical therapy (trunk glide flexion, obstacle avoidance, jumping, combined arm movements, and physical ball games [kicking balls, pushing balls, etc]). CG: Back extensor strengthening exercises+physical therapy.	3 sessions per week 4 weeks	VAS^g^ ODI	4 weeks
Eccleston et al (2022) [43]	United Kingdom	RCT	EG: 14; 17 CG: 11	Male: 5 Female: 37	EG: 55.14; 52.76 CG: 57.09	≥6	EG1: Cognitive behavioral therapy–based immersive VR experience, including virtual mentor-guided “fruit picking” activities, gross motor skills, set in a summer cabin (indoor) and lakeside woods (outdoor). EG2: Sham VR group, using the same VR equipment but displaying only a nontherapeutic 3D seaside environment. CG: Standard care as usual.	15‐60 minutes per day 5 days per week 6‐8 weeks	ODI TSK^h^ NRS^i^	8 weeks 5 months
Garcia et al (2022) [44]	United States	RCT	EG: 89 CG: 90	Male: 41 Female: 137	EG: 52.1 CG: 51.3	≥6	EG: Multimodal VR program based on cognitive behavioral therapy, mindfulness, and pain neuroscience education, including pain education, relaxation training, mindfulness escape scenarios, pain distraction games, and dynamic breathing biofeedback training. CG: Nonimmersive 2D nature scene videos (eg, wildlife documentaries).	2‐16 minutes per day 56 days	DVPRS^j^	8 weeks 3 months 6 months 12 months 18 months 24 months
Hsieh et al (2025) [45]	China	RCT	EG: 35 CG: 35	Male: 26 Female: 44	EG: 61.26 CG: 58.03	EG: 30.83 CG: 42.83	EG: VR-based exergames+physical therapy. Based on LongGood PAPA MAMA system, Ten-full (trunk lateral flexion movements), Taichi (Tai Chi coordination), Doggie Run (dynamic balance training); Adjunctive therapy: 20-minute hot pack+20-minute transcutaneous electrical nerve stimulation. CG: Traditional training includes warm-up, core muscle strengthening, and spinal muscle stretches.	15 minutes per day 3 days per week 2 weeks	ODI	1 week 2 weeks 1 month 3 months
Kim et al (2014) [46]	Korea	RCT	EG: 15 CG: 15	Male: 0 Female: 30	EG: 44.33 CG: 50.46	≥2	EG: Using the Wii Fit balance board for 7 yoga poses (eg, Half Moon, Warrior pose, and Tree pose). CG: Trunk stabilization training, including transversus abdominis or multifidus contraction training, bridge, and plank+conventional physical therapy.	30 minutes 12 times 4 weeks	VAS ODI	4 weeks
Li et al (2021) [47]	China	RCT	EG: 11; 12 CG: 11	Male: 9 Female: 25	EG: 21.91; 23.75 CG: 25.36	EG: 30.18; 38.83 CG: 49.82	EG1: VR-based exergames+physical therapy. Kinect Xbox 360 system for *FruitNinja* game training (required to avoid trunk flexion, cutting virtual fruits, and avoiding bombs using arm movements only). EG2: Ultrasound-guided abdominal drawing-in maneuver and quadruped exercises performed on top of magnetotherapy. CG: Magnetotherapy only.	30 minutes per day 5 days per week 2 weeks	VAS ODI	2 weeks
Massah et al (2025) [49]	Iran	RCT	EG: 20 CG: 20	Male: 22 Female: 17	EG: 31.15 CG: 30.85	≥3	EG: Using the Xbox 360 Kinect for 5 types of VR exergames (River Rush, Rally Ball, etc) to activate postural control muscles. CG: Core stability training performed on a yoga mat (transversus abdominis activation, bridge, plank, etc), emphasizing lumbopelvic neutral position.	30‐45 minutes	TSK VAS	—
Matheve et al (2020) [50]	Belgium	RCT	EG: 42 CG: 42	Male: 30 Female: 54	EG: 42.1 CG: 44.2	CG: 10.6 EG: 10.8	EG: Performing pelvic tilt movements controlled via nonimmersive VR games (ValedoPro sensor) to control virtual characters (eg, caterpillar and fish) to achieve targets. CG: Same pelvic tilt movements, but rhythm controlled by metronome sound, without VR equipment.	4 minutes	NPRS^k^ TSK	—
McConnell et al (2024) [51]	United States	RCT	EG: 19 CG: 13	Male: 12 Female: 20	EG: 48.2 CG: 43.3	≥3	EG: VR-PNE^l^: Delivered via PICO G2 4K headset, containing 12 modules covering pain neuroscience education, mindfulness training, breathing exercises, and patient cases. CG: Conventional physical therapy (PT as usual): Treatment plan formulated by physiotherapists based on clinical routine (including exercise and manual therapy).	21 minutes per time ≥6 times 6 weeks	ODI NRS	6 weeks
Meinke et al (2022) [52]	Switzerland	RCT	EG: 13 CG: 14	Male: 10 Female: 17	EG: 40.14 CG: 40.85	—	EG: VR-based exergames via 2 inertial measurement units (for lumbopelvic movement control, classified as pelvic tilt movements). CG: Usual care.	20 minutes per session 3 sessions per week 3 weeks	NRS TSK	3 weeks
Nambi et al (2020) [54]	Saudi Arabia	RCT	EG: 15; 15 CG: 15	Male: 45	EG: 21.25; 20.23 CG: 20.78	EG: 4.1; 4.1 CG: 4.3	EG1: VR balance training based on the Pro-Kin system (shooting game and core stability training). EG2: Balance training using a Swiss ball. CG: Conventional balance training via active isotonic and isometric exercise.	20 sessions 5 times per week 30 minutes per session	VAS	4 weeks 8 weeks 6 months
Nambi et al (2021) [65]	Saudi Arabia	RCT	EG: 20; 20 CG: 20	Male: 60	EG: 21.45; 20.39 CG: 20.97	EG: 4.8; 5.2 CG: 4.9	EG1: VR training via a shooting game. EG2: Core stability training using a therapy ball. CG: Traditional active balance exercises.	20 sessions 5 times per week 30 minutes per session	NRS	4 weeks 8 weeks 6 months
Nambi et al (2021) [53]	Saudi Arabia	RCT	EG: 20; 20 CG: 20	Male: 60	EG: 23.2; 22.8 CG: 23.3	EG: 5.8; 5.2 CG: 5.4	EG1: VR training+hot pack therapy+ultrasound. EG2: Isokinetic training+hot pack therapy+ultrasound. CG: Conventional core training+hot pack therapy+ultrasound.	20 sessions 5 times per week 30 minutes per session	VAS TSK	4 weeks 6 months
Tuck et al (2022) [56]	New Zealand	RCT	EG: 10 CG: 10; 6	Male: 7 Female: 13	40.1	≥12	EG: Active VR intervention involving games encouraging whole-body movement. CG1: 6-week no-intervention waiting period (waitlist group). CG2: Treatment as usual group (pain neuroscience education, home exercise program, and graded activity exposure).	2 times per week 6 weeks	TSK	6 weeks
Yilmaz Yelvar et al (2017) [58]	Iran	RCT	EG: 22 CG: 22	Male: 16 Female: 28	EG: 46.27 CG: 52.81	EG: 5.27 CG: 7.45	EG: Completing virtual walking tasks via Vita Digital Productions software+physical therapy. CG: Physical therapy (eg, hot pack, transcutaneous electrical nerve stimulation, ultrasound, deep heat therapy, and therapeutic exercise).	10 sessions 5 times per week	VAS TSK ODI	2 weeks
Zadro et al (2019) [59]	Turkey	RCT	EG: 30 CG: 30	Male: 29 Female: 31	EG: 68.8 CG: 67.8	>3	EG: Video game home workout (mixed exercise including aerobic, resistance, and yoga). CG: Daily life routines.	24 sessions 3 times per week 60 minutes per session	NRS TSK	8 weeks
Oh et al (2014) [61]	South Korea	RCT	EG: 9; 9; 10 CG: 9	Male: 37	EG: 20.7; 20.56; 20.33 CG: 20.44	EG: 6.38; 6.21; 7.57 CG: 6.75	EG: 10-minute VR training via horse riding simulator; 20-minute VR training via horse riding simulator; and 30-minute VR training via horse riding simulator. CG: Daily life routines.	40 sessions 5 times per week 10, 20, and 30 minutes per session	VAS	8 weeks
Park et al (2013) [62]	South Korea	RCT	EG: 8; 8 CG: 8	—	EG: 44.12; 43.37 CG: 44.12	EG: 17.0; 16.0 CG: 18.75	EG1: VR training (multiple games)+physical therapy. EG2: Lumbar stabilization exercises (classified as core stability training)+physical therapy. CG: Physical therapy (eg, hot pack, interferential current therapy, and ultrasound deep heat therapy).	24 sessions 3 times per week 80 minutes per session	VAS	8 weeks
Park et al (2020) [63]	South Korea	RCT	EG: 40 CG: 40	Female: 80	EG: 71.35 CG: 72.05	EG: 23.61 CG: 22.10	EG: VR-based simulated horseback riding training. CG: Sitting on the horse riding simulator, without VR or active training.	36 sessions 3 times per week 30 minutes per session	VAS ODI	12 weeks
Yoo et al (2014) [64]	South Korea	RCT	EG: 24 CG: 23	Male: 47	EG: 20.44 CG: 20.7	EG: 9.41 CG: 8.35	EG: VR-based horse simulation training. CG: Daily life routines.	24 sessions 3 times per week 20‐50 minutes per session	VAS	8 weeks
Kim et al (2020) [60]	South Korea	RCT	EG: 24 CG: 24	Male: 26 Female: 22	EG: 26.0 CG: 28.79	CG: 101.55 EG: 58.22	EG: VR-based simulated horse riding. CG: Stabilization exercises using sling suspension.	16 sessions 2 times per week 46 minutes per session	NRS ODI	4 weeks 8 weeks 6 months
Yalfani et al (2022) [57]	Iran	RCT	EG: 13 CG: 12	Female: 25	EG: 68 CG: 67.08	≥6	EG: HTC Vive VR system, 8 games (eg, boxing, soccer, and skiing involving strength, balance, coordination, and cognition), combining upper limb, lower limb, and trunk synergistic movements. CG: No specific treatment, maintaining daily life activities only.	24 sessions 3 times per week 30 minutes per session	VAS	2 weeks
Sato et al (2021) [55]	Japan	RCT	EG: 20 CG: 20	Male: 21 Female: 19	EG: 49.3 CG: 55.6	>3	EG: Training using Nintendo RFA^m^ exergame (combining aerobic exercises like jogging, squats, resistance training, and yoga or stretches targeting the lower back), including Adventure Mode (30 minutes)+Lower Back Pain Improvement Program (10 minutes). CG: Continuing original drug treatment (unchanged dosage).	40 minutes per session 1 session per week 8 weeks	VAS TSK	8 weeks
Maddox et al (2023) [48]	United States	RCT	EG: 536 CG: 531	Female: 772 Male: 293	EG: 50.4 CG: 51.1	≥3	EG: Diaphragmatic breathing training, cognitive-emotional regulation mindfulness training, and pain education. CG: Sham VR: 20-segment looped nature scenery videos displayed on a large screen with background music.	2‐16 minutes per day 56 days	ODI	8 weeks 1, 2, 3, 6, 12, 18, and 24 months

a RCT: randomized controlled trial.

b EG: experimental group.

c CG: control group.

d ODI: Oswestry Disability Index.

e VR: virtual reality.

f Not available.

g VAS: visual analog scale.

h TSK: Tampa Scale of Kinesiophobia.

i NRS: Numerical Rating Scale.

j DVPRS: Defense and Veterans Pain Rating Scale.

k NPRS: Numerical Pain Rating Scale.

l RFA: Ring Fit Adventure.

m VR-PNE: Virtual Reality Pain Neuroscience Education.

### Risk of Bias Assessment

**Figure 2.**
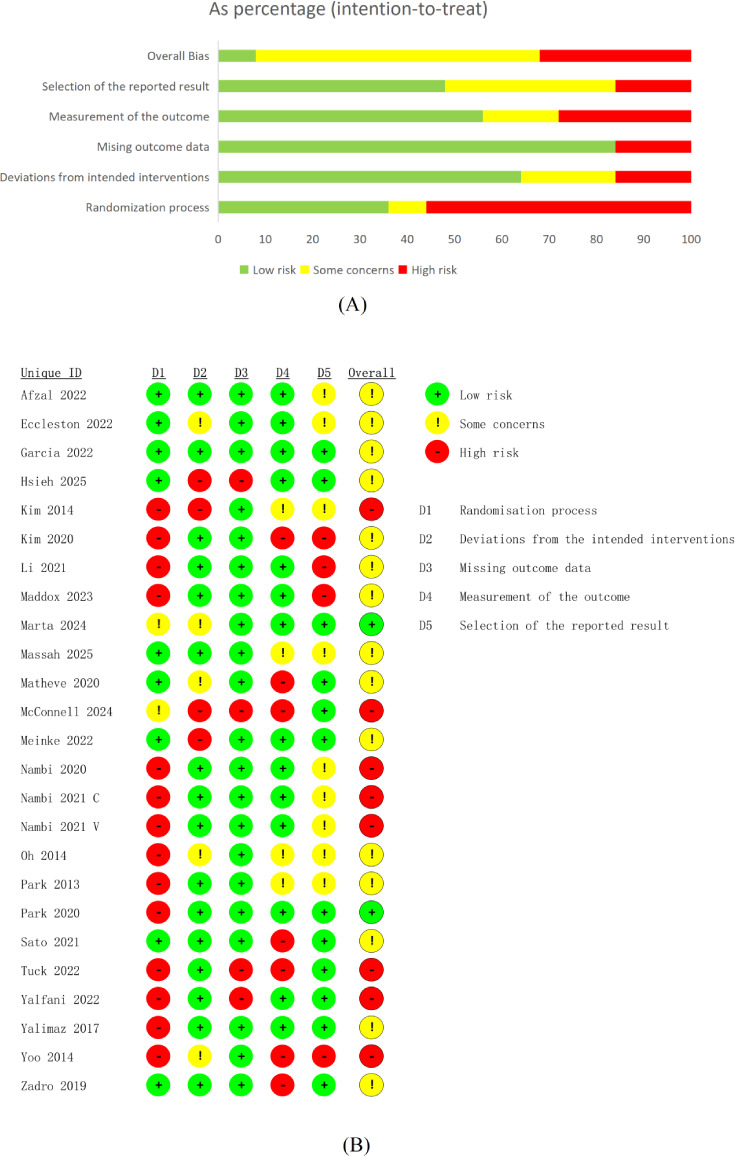
Assessment results of Risk of Bias 2: (A) risk of bias graph and (B) risk of bias summary [41-65].

The methodological quality of the included RCTs was assessed using the Cochrane Risk of Bias 2 tool. Among the 25 studies, 2 were rated as low risk, 15 as moderate risk, and 8 as high risk (Figure 2). Overall, the methodological quality of the included studies was judged as moderate. The interrater agreement κ values for each domain ranged from 0.61 to 0.84, reflecting substantial consistency among assessors.

### Strength of Evidence

The quality of evidence across the outcomes varied. For pain intensity (visual analog scale), it ranged from moderate to very low, with most comparisons rated as low or very low. All comparisons for disability and kinesiophobia were rated as very low. The downgrading of evidence was primarily due to within-study bias, imprecision, and heterogeneity or incoherence. Indirectness and reporting bias were not reasons for downgrading in any comparison. The results of all comparisons are provided in Table 3.

**Table 3.** Summary table credibility assessment using CINeMA^a^.

Comparison	Studies, n	Within-study bias	Reporting bias	Indirectness	Imprecision	Heterogeneity	Incoherence	Confidence rating
Pain intensity								
CON1^b^:VRBHj^c^	3	Major concerns	Low risk	No concerns	No concerns	No concerns	No concerns	Low
CON1:VRBHm^d^	1	Some concerns	Low risk	No concerns	No concerns	Major concerns	Major concerns	Very low
CON1:VRBL^e^	1	Some concerns	Low risk	No concerns	Major concerns	No concerns	No concerns	Very low
CON1:VRBMix^f^	1	Some concerns	Low risk	No concerns	Major concerns	No concerns	No concerns	Very low
CON1:VRBR^g^	1	Some concerns	Low risk	No concerns	Major concerns	No concerns	No concerns	Very low
CON1:VRBY^h^	1	Major concerns	Low risk	No concerns	Major concerns	No concerns	No concerns	Very low
CON2:VRBHm^i^	1	Some concerns	Low risk	No concerns	No concerns	Major concerns	No concerns	Very low
CON2:VRBMix	1	Some concerns	Low risk	No concerns	Major concerns	No concerns	No concerns	Very low
CON2:VRBO^j^	1	Some concerns	Low risk	No concerns	Major concerns	No concerns	No concerns	Very low
CON2:VRBR	2	Some concerns	Low risk	No concerns	Major concerns	No concerns	No concerns	Very low
CON3:VRBHm	2	Some concerns	Low risk	No concerns	No concerns	Major concerns	No concerns	Very low
CON3:VRBL^k^	1	Some concerns	Low risk	No concerns	Major concerns	No concerns	No concerns	Very low
CON3:VRBMix	3	Some concerns	Low risk	No concerns	No concerns	Major concerns	No concerns	Very low
CON3:VRBR	2	Some concerns	Low risk	No concerns	Major concerns	No concerns	No concerns	Very low
Indirect evidence								
CON1:VRBO	0	Some concerns	Low risk	No concerns	Major concerns	No concerns	No concerns	Very low
CON2:VRBHj	0	Some concerns	Low risk	No concerns	No concerns	No concerns	No concerns	Moderate
CON2:VRBL	0	Some concerns	Low risk	No concerns	Major concerns	No concerns	No concerns	Very low
CON2:VRBY	0	Some concerns	Low risk	No concerns	Major concerns	No concerns	No concerns	Very low
CON3:VRBHj	0	Some concerns	Low risk	No concerns	No concerns	No concerns	No concerns	Moderate
CON3:VRBO	0	Some concerns	Low risk	No concerns	Major concerns	No concerns	No concerns	Very low
CON3:VRBY	0	Some concerns	Low risk	No concerns	Major concerns	No concerns	No concerns	Very low
VRBHj:VRBHm	0	Some concerns	Low risk	No concerns	No concerns	Major concerns	No concerns	Very low
VRBHj:VRBL	0	Some concerns	Low risk	No concerns	No concerns	No concerns	No concerns	Moderate
VRBHj:VRBMix	0	Some concerns	Low risk	No concerns	No concerns	No concerns	No concerns	Moderate
VRBHj:VRBO	0	Some concerns	Low risk	No concerns	No concerns	Major concerns	No concerns	Very low
VRBHj:VRBR	0	Major concerns	Low risk	No concerns	No concerns	No concerns	No concerns	Low
VRBHj:VRBY	0	Major concerns	Low risk	No concerns	No concerns	Major concerns	No concerns	Very low
VRBHm:VRBL	0	Some concerns	Low risk	No concerns	Major concerns	No concerns	No concerns	Very low
VRBHm:VRBMix	0	Some concerns	Low risk	No concerns	Major concerns	No concerns	No concerns	Very low
VRBHm:VRBO	0	Some concerns	Low risk	No concerns	Major concerns	No concerns	No concerns	Very low
VRBHm:VRBR	0	Some concerns	Low risk	No concerns	No concerns	Major concerns	No concerns	Very low
VRBHm:VRBY	0	Some concerns	Low risk	No concerns	Major concerns	No concerns	No concerns	Very low
VRBL:VRBMix	0	Some concerns	Low risk	No concerns	Major concerns	No concerns	No concerns	Very low
VRBL:VRBO	0	Some concerns	Low risk	No concerns	Major concerns	No concerns	No concerns	Very low
VRBL:VRBR	0	Some concerns	Low risk	No concerns	Major concerns	No concerns	No concerns	Very low
VRBL:VRBY	0	Some concerns	Low risk	No concerns	Major concerns	No concerns	No concerns	Very low
VRBMix:VRBO	0	Some concerns	Low risk	No concerns	Major concerns	No concerns	No concerns	Very low
VRBMix:VRBR	0	Some concerns	Low risk	No concerns	Major concerns	No concerns	No concerns	Very low
VRBMix:VRBY	0	Some concerns	Low risk	No concerns	Major concerns	No concerns	No concerns	Very low
VRBO:VRBR	0	Some concerns	Low risk	No concerns	Major concerns	No concerns	No concerns	Very low
VRBO:VRBY	0	Some concerns	Low risk	No concerns	Major concerns	No concerns	No concerns	Very low
VRBR:VRBY	0	Major concerns	Low risk	No concerns	Major concerns	No concerns	No concerns	Very low
Function								
CON1:VRBHm	1	Some concerns	Low risk	No concerns	Major concerns	No concerns	Major concerns	Very low
CON1:VRBMix	3	Some concerns	Low risk	No concerns	Major concerns	No concerns	Major concerns	Very low
CON1:VRBY	1	Major concerns	Low risk	No concerns	Major concerns	No concerns	Major concerns	Very low
CON2:VRBHm	1	No concerns	Low risk	No concerns	No concerns	Major concerns	Major concerns	Very low
CON2:VRBO	1	Some concerns	Low risk	No concerns	Major concerns	No concerns	Major concerns	Very low
CON2:VRBR	2	Some concerns	Low risk	No concerns	Major concerns	No concerns	Major concerns	Very low
CON3:VRBR	1	Some concerns	Low risk	No concerns	Major concerns	No concerns	Major concerns	Very low
Indirect evidence								
CON1:VRBO	0	Some concerns	Low risk	No concerns	Major concerns	No concerns	Major concerns	Very low
CON1:VRBR	0	Some concerns	Low risk	No concerns	Major concerns	No concerns	Major concerns	Very low
CON2:VRBMix	0	Some concerns	Low risk	No concerns	No concerns	Major concerns	Major concerns	Very low
CON2:VRBY	0	Some concerns	Low risk	No concerns	No concerns	Major concerns	Major concerns	Very low
CON3:VRBHm	0	Some concerns	Low risk	No concerns	No concerns	Major concerns	Major concerns	Very low
CON3:VRBMix	0	Some concerns	Low risk	No concerns	No concerns	Major concerns	Major concerns	Very low
CON3:VRBO	0	Some concerns	Low risk	No concerns	Major concerns	No concerns	Major concerns	Very low
CON3:VRBY	0	Some concerns	Low risk	No concerns	Major concerns	No concerns	Major concerns	Very low
VRBHm:VRBMix	0	Some concerns	Low risk	No concerns	Major concerns	No concerns	Major concerns	Very low
VRBHm:VRBO	0	Some concerns	Low risk	No concerns	No concerns	Major concerns	Major concerns	Very low
VRBHm:VRBR	0	Some concerns	Low risk	No concerns	No concerns	Major concerns	Major concerns	Very low
VRBHm:VRBY	0	Major concerns	Low risk	No concerns	Major concerns	No concerns	Major concerns	Very low
VRBMix:VRBO	0	Some concerns	Low risk	No concerns	No concerns	Major concerns	Major concerns	Very low
VRBMix:VRBR	0	Some concerns	Low risk	No concerns	No concerns	Major concerns	Major concerns	Very low
VRBMix:VRBY	0	Some concerns	Low risk	No concerns	Major concerns	No concerns	Major concerns	Very low
VRBO:VRBR	0	Some concerns	Low risk	No concerns	Major concerns	No concerns	Major concerns	Very low
VRBO:VRBY	0	Some concerns	Low risk	No concerns	Major concerns	No concerns	Major concerns	Very low
VRBR:VRBY	0	Some concerns	Low risk	No concerns	Major concerns	No concerns	Major concerns	Very low
Kinesiophobia								
CON1:VRBHj	1	Major concerns	Low risk	No concerns	No concerns	Major concerns	Major concerns	Very low
CON1:VRBMix	1	Some concerns	Low risk	No concerns	No concerns	Major concerns	Major concerns	Very low
CON3:VRBL	1	Some concerns	Low risk	No concerns	Major concerns	No concerns	Major concerns	Very low
CON3:VRBMix	3	Some concerns	Low risk	No concerns	Major concerns	No concerns	Major concerns	Very low
CON3:VRBR	1	Some concerns	Low risk	No concerns	Major concerns	No concerns	Major concerns	Very low
Indirect evidence								
CON1:VRBL	0	Some concerns	Low risk	No concerns	No concerns	Major concerns	Major concerns	Very low
CON1:VRBR	0	Some concerns	Low risk	No concerns	No concerns	Major concerns	Major concerns	Very low
CON3:VRBHj	0	Some concerns	Low risk	No concerns	No concerns	Major concerns	Major concerns	Very low
VRBHj:VRBL	0	Some concerns	Low risk	No concerns	No concerns	Major concerns	Major concerns	Very low
VRBHj:VRBMix	0	Major concerns	Low risk	No concerns	No concerns	Major concerns	Major concerns	Very low
VRBHj:VRBR	0	Some concerns	Low risk	No concerns	No concerns	Major concerns	Major concerns	Very low
VRBL:VRBMix	0	Some concerns	Low risk	No concerns	Major concerns	No concerns	Major concerns	Very low
VRBL:VRBR	0	Some concerns	Low risk	No concerns	Major concerns	No concerns	Major concerns	Very low
VRBMix:VRBR	0	Some concerns	Low risk	No concerns	Major concerns	No concerns	Major concerns	Very low

a CINeMA: Confidence in Network Meta-Analysis.

b CON1: traditional exercise control.

c VRBHj: shooting game.

d VRBHm: VR-based equestrian training.

e VRBL: pelvic flexibility game.

f VRBMix: multicomponent mixed game.

g VRBR: VR-based cognitive behavioral therapy.

h VRBY: VR-based yoga training.

i CON2: nonexercise control.

j VRBO: VR-based walking training.

k CON3: placebo control.

### Network Meta-Analysis Results

#### Effect on Pain Intensity

**Figure 3.**
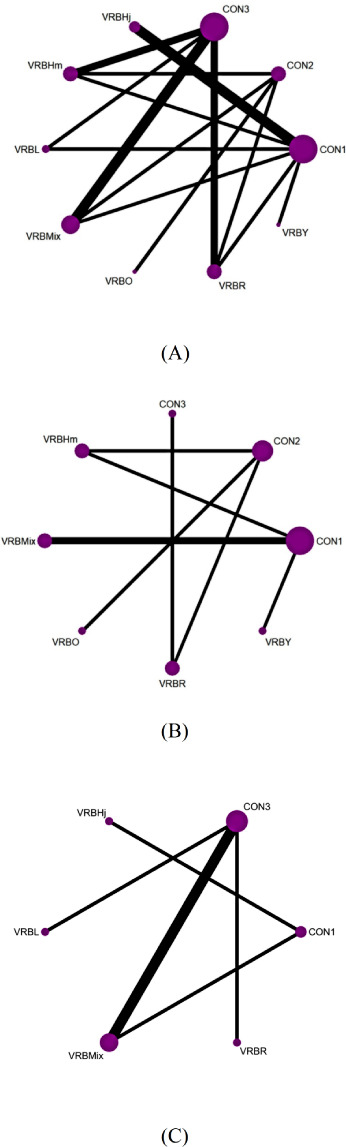
Network plot of different outcome measures. (A) Pain intensity, (B) disability (Oswestry Disability Index), and (C) kinesiophobia (Tampa Scale of Kinesiophobia). CON1: traditional exercise control; CON2: nonexercise control; CON3: placebo control; VRBHj: shooting game; VRBHm: VR-based equestrian training; VRBL: pelvic flexibility game; VRBO: VR-based walking training; VRBR: VR-based cognitive behavioral therapy; VRBY: VR-based yoga training; VRBMix: multicomponent mixed game.

A total of 1017 patients from 21 studies were included to evaluate the efficacy of different VR exposure therapies in alleviating CLBP. In total, 7 interventions and 3 control measures were analyzed. The network relationship diagram is detailed in Figure 3.

**Figure 4.**
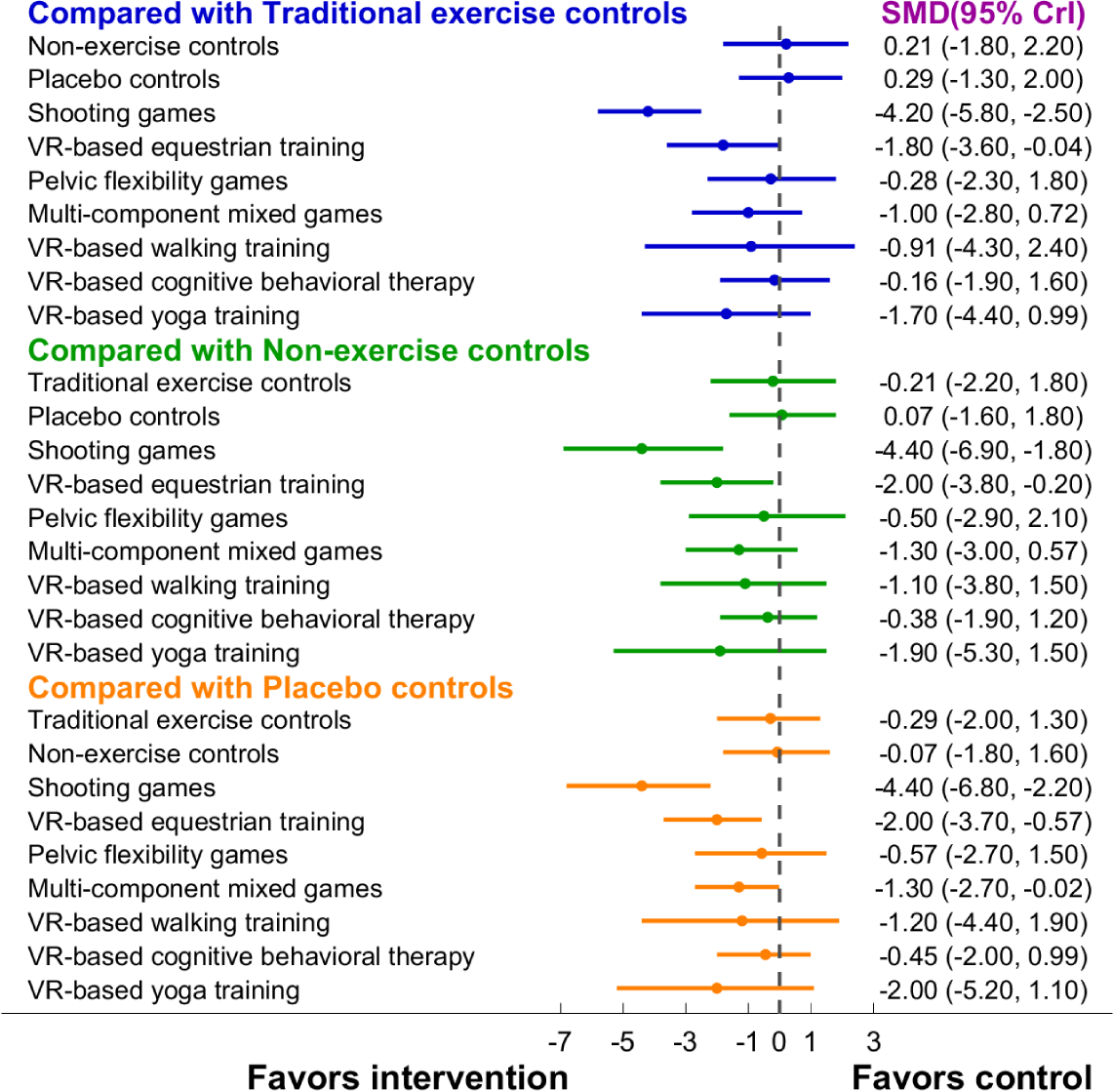
Forest plot for pairwise comparisons of pain intensity. Interpretation: each row represents a comparison between an intervention (listed on the Y axis) and the reference control. The square marks the point estimate of the SMD, and the horizontal line represents the 95% CrI. The vertical dashed line at SMD=0 indicates no effect. Comparisons where the entire CrI lies to the left of this line (SMD <0) indicate that the intervention is statistically superior to the control. For example, “shooting games” shows a CrI entirely below 0, signifying a significant reduction in pain compared to placebo. The surface under the cumulative ranking curve is provided in Figure 5. CrI: credible interval; SMD: standardized mean difference; VR: virtual reality.

**Figure 5.**
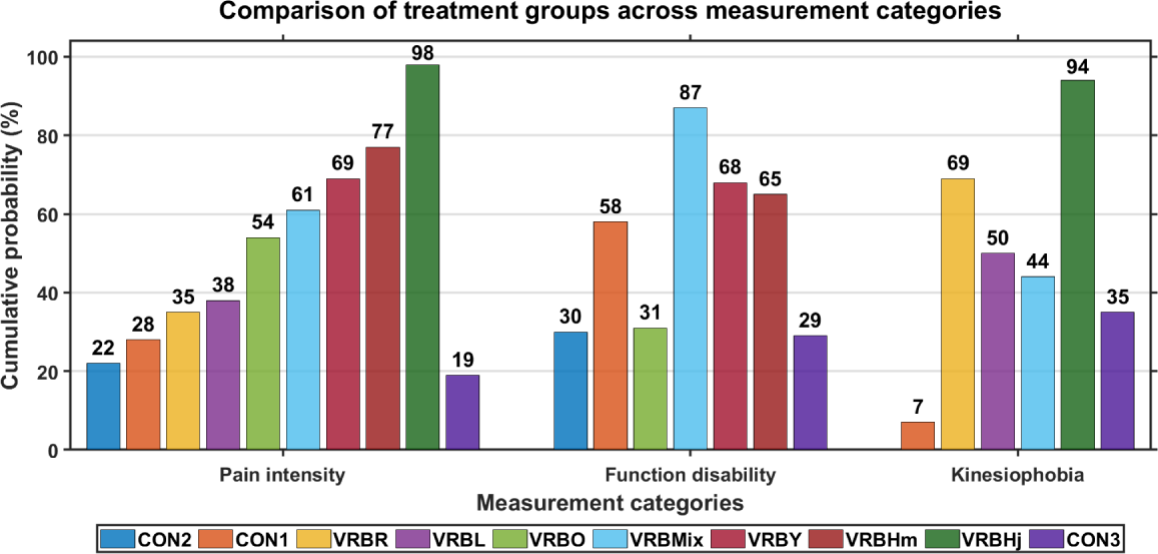
Rank heat plot of the different outcomes analyzed. Numbers and colors indicate the probability of being the first intervention recommended for a certain outcome. CON1: traditional exercise control; CON2: nonexercise control; CON3: placebo control; VRBHj: shooting game; VRBHm: VR-based equestrian training; VRBL: pelvic flexibility game; VRBO: VR-based walking training; VRBR: VR-based cognitive behavioral therapy; VRBY: VR-based yoga training; VRBMix: multicomponent mixed game.

Forest plot results indicated that shooting games (SMD −4.40, 95% CrI −6.80 to −2.20), VR-based equestrian training (SMD −2.00, 95% CrI −3.70 to −0.57), and multicomponent mixed games (SMD −1.30, 95% CrI −2.7 to −0.027) were significantly superior to placebo controls (Figure 4). Shooting games (traditional exercise control: SMD −4.20, 95% CrI −5.80 to −2.50; nonexercise control: SMD −4.40, 95% CrI −6.90 to −1.80) and VR-based equestrian training (traditional exercise control: SMD −1.80, 95% CrI −3.60 to −0.044; nonexercise control: SMD −2.00, 95% CrI −3.80 to −0.20) demonstrated significantly superior efficacy compared to traditional exercise controls and nonexercise controls. Detailed effect sizes and CrI for pairwise comparisons between interventions were presented in the league table. Specifically, shooting games were significantly more effective than traditional exercise controls (SMD 4.16, 95% CrI 2.53-5.83), nonexercise controls (SMD 4.37, 95% CrI 1.81-6.94), placebo controls (SMD 4.45, 95% CrI 2.17-6.86), pelvic flexibility games (SMD −3.88, 95% CrI −6.56 to −1.32), VR-based cognitive behavioral therapy (SMD −3.99, 95% CrI −6.44 to −1.63), and multicomponent mixed games (SMD −3.13, 95% CrI −5.56 to −0.78). SUCRA results (Figure 5 and Figure S2, Multimedia Appendix 2) indicated that shooting games were most likely to serve as a first-line intervention (98%), followed by VR-based equestrian training (77%), VR-based yoga training (69%), and multicomponent mixed games (60%).

Consistency modeling indicated substantial overall network heterogeneity (*I*^2^=87.6%). Network meta-regression revealed no significant moderating effects of covariates. Consistency between the consistency and inconsistency models was good, and node-splitting analysis (Figure S4, Multimedia Appendix 2) found no significant discrepancies between direct and indirect evidence across comparisons (*P*>.05). The consistency model converged well, and all studies met the model-fit criteria, obviating sensitivity analysis. Adjusted funnel plots showed a symmetrical distribution and nonsignificant Egger tests (*P*=.84), indicating no substantial publication bias (Figure S3, Multimedia Appendix 2).

#### Effect on Disability (Oswestry Disability Index)

To assess the impact of various VR exposure therapies on reducing disability in individuals with CLBP, 1485 participants from 10 studies were included. The comparison results between the 3 control groups and 5 interventions were displayed in the network diagram (Figure 3).

**Figure 6.**
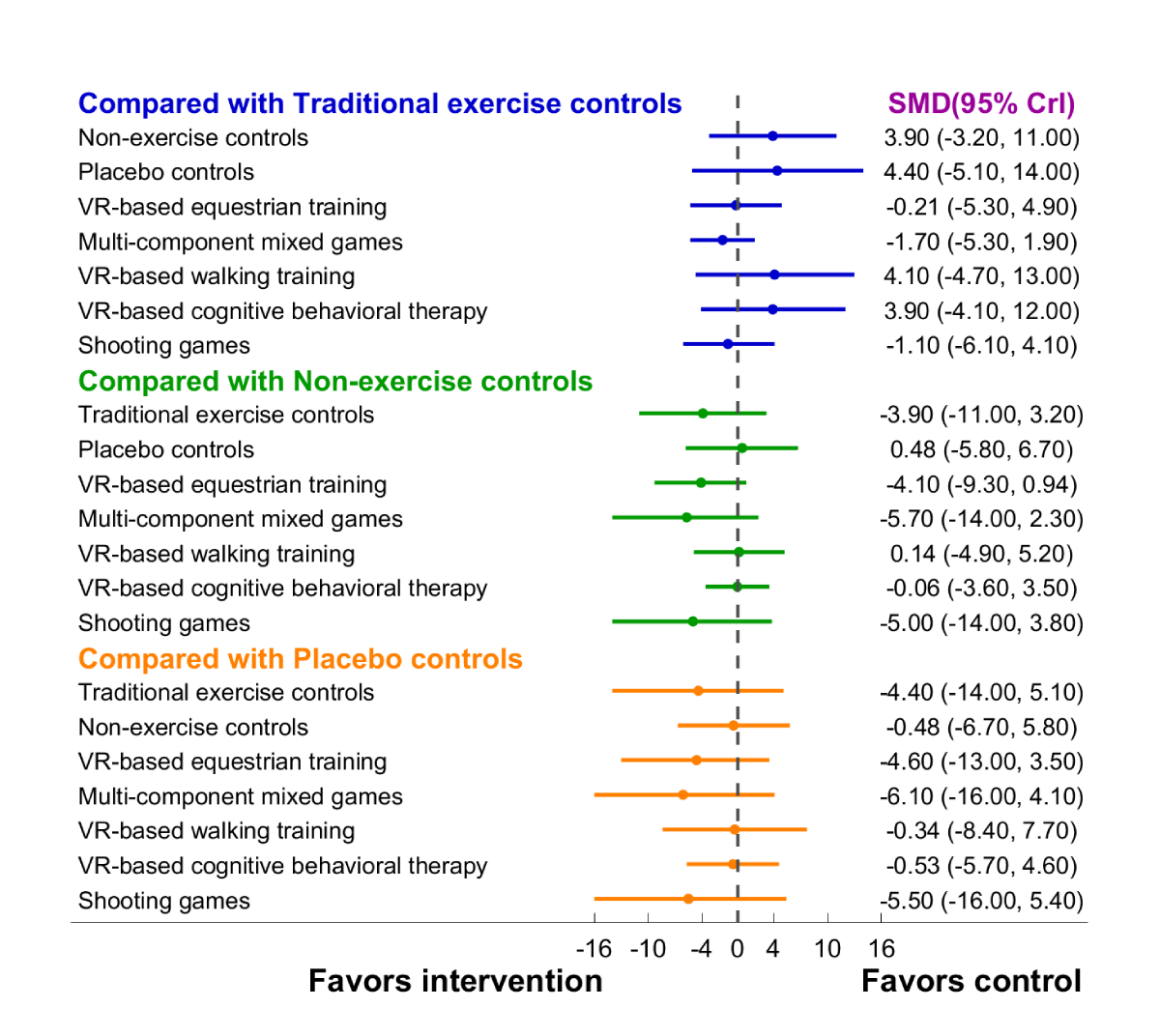
Forest plot for pairwise comparisons of disability. CrI: credible interval; SMD: standardized mean difference; VR: virtual reality.

The results showed that none of the interventions led to significant improvement in disability, with no significant differences observed across all pairwise comparisons (Figure 6 and Figure S1, Multimedia Appendix 2). Overall heterogeneity was substantial (*I*^2^=96.6%), and it was exceptionally high (*I*^2^=98.3%) when comparing multicomponent mixed games to traditional exercise controls pairwise (Figure S5, Multimedia Appendix 2). Multicomponent mixed games were scored best (84%), followed by VR-based yoga training (75%) and VR-based equestrian training (70%), as viewed in the cumulative probability ranking data (Figure 5). Covariates did not significantly affect treatment effects, observed in network meta-regression analysis. The results of consistent and inconsistent models were comparable. As shown by the convergence diagnostics, all studies fit the model correctly. The corrected funnel plot showed that the majority of the studies had a symmetrical distribution, indicating that there was no chance of publication bias (Figure S3, Multimedia Appendix 2).

#### Effect on Kinesiophobia (Tampa Scale of Kinesiophobia)

In total, 7 studies involving 250 patients were included to analyze the efficacy of different VR exposure therapies in reducing kinesiophobia in patients with CLBP, including 4 interventions and 2 control groups (Figure 3).

**Figure 7.**
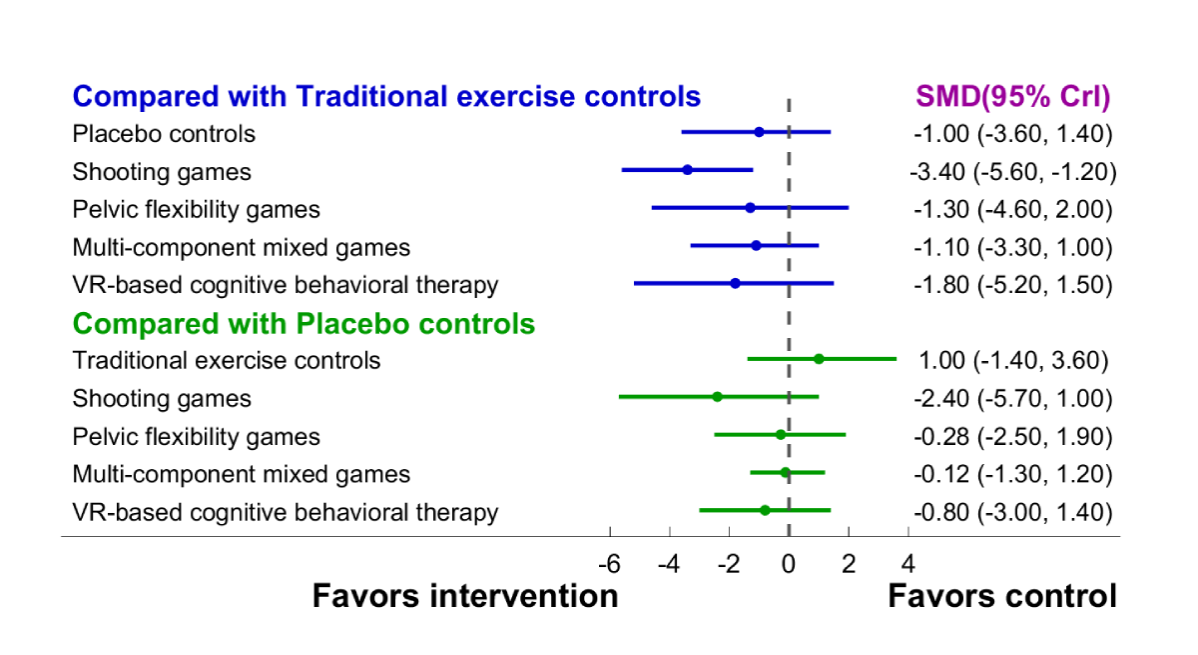
Forest plot for pairwise comparisons of kinesiophobia. CrI: credible interval; SMD: standardized mean difference; VR: virtual reality.

Figure 7 presents that shooting games demonstrated specific advantages in reducing kinesiophobia, showing statistically significant superiority over traditional exercise controls (SMD −3.40, 95% CrI −5.60 to −1.20). Ranking results (Figure 5) placed shooting games first (94%), followed by VR-based cognitive behavioral therapy (69%).

There was little heterogeneity (*I*^2^=0.00%), indicating that the studies included in this analysis exhibited minimal variability with each other. Overall model fit was good. Convergence diagnostics confirmed that all investigations fit the model correctly. The corrected funnel plot showed that the majority of the studies had a symmetrical distribution, indicating that there was no chance of publication bias (Figure S3, Multimedia Appendix 2).

## Discussion

### Principal Findings

Consistent with previous systematic reviews, the present network meta-analysis demonstrated that shooting games and VR-based equestrian training significantly reduced pain intensity in patients with CLBP. No VR intervention yielded a statistically significant improvement in disability. Shooting games significantly outperformed traditional exercise controls in reducing kinesiophobia, although no significant difference was observed relative to nonexercise or placebo controls. Based on the GRADE (Grading of Recommendations Assessment, Development and Evaluation) criteria, the overall quality of evidence ranged from low to very low, primarily due to high heterogeneity, risk of bias, and imprecision. Network meta-regression revealed no significant moderating effects of covariates. Despite significant heterogeneity, the good consistency of the model supports the reference value of the present findings [66].

The superior performance of shooting games and VR-based equestrian training in pain intensity may be explained by their integration of core stability training with cognitive-behavioral elements within an immersive, interactive environment [67]. The shooting game intervention was not blinded to participants or outcome assessors throughout implementation, which could have resulted in an overestimation of the treatment effect; this conclusion should be regarded cautiously [68]. Exercise therapy and cognitive behavioral treatment are 2 examples of how pain science is moving toward a biopsychosocial model. By strengthening movement confidence, encouraging graded exposure to movement, and improving attentional distraction, these modalities probably address both the nociceptive and emotional aspects of pain [69]. The combination of physical and psychological involvement may be essential for therapeutic success, as seen by the lower rankings for pain outcomes of therapies that mostly rely on cognitive restructuring or passive distraction, such as VR-based cognitive behavioral therapy. None of the VR therapies significantly improved impairment. Moreover, the certainty of evidence for all disability comparisons was rated as very low, due to serious imprecision, inconsistency, and high risk of bias. There is a possibility that excessive clinical heterogeneity between studies may have led to nonsignificant results [70]; therefore, we searched for another disability assessment tool for comparison. The studies by Kim et al [60] and Zadro et al [59] used the Roland-Morris Questionnaire for disability assessment, and both reported no statistically significant differences between groups, which further supports the conclusions of this study. The analysis of Li et al [22] showed notable gains in the disability index, most likely as a result of selecting possibly more successful trials by establishing a criterion of ≥8 interventions. In contrast, traditional exercise therapy leads to more significant improvement in disability [71]. While VR interventions frequently concentrate on improving engagement and adherence without clearly separating the “medium effect” from the “therapeutic component effect,” traditional interventions benefit from well-validated mechanisms that directly target functional capacity. This could help to explain why the evidence for disability outcomes is still far less certain than that for pain intensity [72]. We found a decrease in kinesiophobia that was comparable to the improvement trend in pain scores when comparing the shooting game to conventional exercise therapy. This discrepancy may be caused by the lack of diversion, which increases kinesiophobia levels during treatment [10]. However, this comparison is based on data from a single study and has very poor-quality evidence. We summarized the characteristics of the intervention in this study, where participants were exclusively soccer athletes with an average age below 30 years. The unique physical activity levels, beliefs, and attitudes toward exercise within this population may introduce confounding factors affecting the reliability. This population’s particular levels of physical activity, attitudes toward exercise, and ideas about it could introduce confounding factors that alter the reliability [54,65].

### Compared With Other Reviews

The present findings are consistent with and extend previous evidence syntheses. Huang et al [16] reported no significant analgesic effect of VR for CLBP, but their analysis included only 2 RCTs with limited statistical power. Kumar et al [24] and Choi et al [25] also concluded that VR treatment can reduce pain; they did not perform subgroup analyses based on VR game type, level of immersion, or degree of physical involvement, nor did they explore how different game contents might influence outcomes. Li et al [22] confirmed that VR training improves pain, kinesiophobia, and disability immediately after the intervention, but did not distinguish specific VR game types. Lo et al [18] suggested that nonimmersive VR is more effective for low back pain, while Henríquez-Jurado et al [23] found no significant difference between immersive and nonimmersive VR. These studies indicate that hardware-based classification fails to capture therapeutic differences.

Brea-Gómez et al [11] conducted a meta-analysis stratified by VR devices. By performing subgroup analyses based on 3 distinct types of VR devices (Nintendo consoles, horse-riding simulators, and ProKin system), the study found that horse-riding simulators and the ProKin system significantly reduced pain compared with control conditions, whereas Nintendo consoles did not show a significant effect. Regarding kinesiophobia, the ProKin system was effective, while Nintendo consoles even yielded unfavorable results. These findings represent an important early effort to explore the differential efficacy of various VR game-like devices, clearly indicating that hardware characteristics and interaction modalities including sensor precision, immersion level, and task type may critically influence treatment outcomes. Rather than classifying VR interventions based on hardware or immersion level, this study extends research on efficacy differences across VR categories by refining task‐specific typologies and directly comparing 7 distinct VR content modalities. We confirmed that VR interventions combining dynamic full-body movement and cognitive engagement (shooting games and equestrian training) are superior to passive or single-task VR modalities. Consistent with previous studies, no VR intervention significantly improved disability, providing more precise network meta-analytic evidence.

### Strengths and Limitations

This study represents the first network meta-analysis to systematically compare and rank the efficacy of distinct VR-based training modalities on pain intensity, disability, and kinesiophobia in patients with CLBP, which moves beyond aggregate categorizations of VR and enables direct and indirect comparisons across different intervention types. By identifying the relative ranking of interventions, this work contributes to the field by clarifying which VR modalities may offer the greatest therapeutic potential and by revealing critical gaps in the existing evidence base, such as high heterogeneity and limited evidence for disability outcomes. However, several limitations warrant consideration. The analysis was constrained by the inclusion of only 25 studies and 7 intervention types. Data were particularly scarce for kinesiophobia, potentially affecting the robustness of network meta-analysis. Furthermore, interventions like pelvic flexibility games, VR-based walking training, and VR-based yoga training are likely undervalued due to limited studies, small samples, wide CIs, and poor network connectivity, reliant on indirect comparisons [73]. The occurrence of VR-related adverse effects and participant dropouts in some studies may limit engagement levels and could affect the interpretation of overall outcomes, even where reported attrition is low.

### Clinical Implications and Future Research

In clinical practice, for the treatment of pain and kinesiophobia using VR, we recommend shooting games, while taking into account the patient’s individual circumstances, or using VR as an adjunctive therapy in combination with other methods. Given the current evidence profile, none of the VR-mediated interventions can be recommended, as determined by substantial heterogeneity, high risk of bias, and low certainty of evidence. Future research could focus on the following. First, to obtain higher-level comparison effect estimates, future research should concentrate on performing more head-to-head RCTs that directly compare various VR exposure regimens. Second, to minimize heterogeneity and improve the reproducibility of outcomes, the hardware components, software components, dosage of the intervention, and length of treatment should be precisely specified. Third, long-term follow-up should be included in studies to evaluate the sustainability of the intervention and investigate its long-term benefits for relapse prevention and improvement of disability.

### Conclusions

Overall, certain VR-based trainings show promise for CLBP. The shooting game demonstrated a relative advantage over the also effective simulated horse-riding training in improving pain and alleviating kinesiophobia, ranking higher in pain control probability. However, definitive conclusions remain limited by insufficient evidence and considerable clinical heterogeneity. More high-quality RCTs are needed to evaluate their short- and long-term benefits and to provide robust evidence.

## Supplementary material

10.2196/90289 Multimedia Appendix 1 Search strategies used in each database.

10.2196/90289 Multimedia Appendix 2 Summary of results of included studies and other data from the network meta‑analysis.

10.2196/90289 Checklist 1 PRISMA 2020 checklist.

10.2196/90289 Checklist 2 PRISMA-S checklist.

## Abbreviations

CINeMA: Confidence in Network Meta-Analysis
CLBP: chronic low back pain
CrI: credible interval
GRADE: Grading of Recommendations Assessment, Development and Evaluation
PRISMA: Preferred Reporting Items for Systematic Reviews and Meta-Analyses
PRISMA-S: Preferred Reporting Items for Systematic Reviews and Meta-Analyses Literature Search Extension
RCT: randomized controlled trial
SMD: standardized mean difference
SUCRA: surface under the cumulative ranking curve
VR: virtual reality

## References

[1] van Tulder M, Becker A, Bekkering T (2006). Chapter 3. European guidelines for the management of acute nonspecific low back pain in primary care. Eur Spine J.

[2] Koes BW, van Tulder MW, Thomas S (2006). Diagnosis and treatment of low back pain. BMJ.

[3] Airaksinen O, Brox JI, Cedraschi C (2006). Chapter 4. European guidelines for the management of chronic nonspecific low back pain. Eur Spine J.

[4] Maher C, Underwood M, Buchbinder R (2017). Non-specific low back pain. Lancet.

[5] GBD 2021 Low Back Pain Collaborators (2023). Global, regional, and national burden of low back pain, 1990-2020, its attributable risk factors, and projections to 2050: a systematic analysis of the Global Burden of Disease Study 2021. Lancet Rheumatol.

[6] Briggs AM, Sumi Y, Banerjee A (2025). The World Health Organization guideline for non-surgical management of chronic primary low back pain in adults: implications for equitable care and strengthening health systems globally. Glob Health Res Policy.

[7] Oliveira CB, Maher CG, Pinto RZ (2018). Clinical practice guidelines for the management of non-specific low back pain in primary care: an updated overview. Eur Spine J.

[8] Borsook D, Youssef AM, Simons L, Elman I, Eccleston C (2018). When pain gets stuck: the evolution of pain chronification and treatment resistance. Pain.

[9] Rogers AH, Farris SG (2022). A meta-analysis of the associations of elements of the fear-avoidance model of chronic pain with negative affect, depression, anxiety, pain-related disability and pain intensity. Eur J Pain.

[10] Alaiti RK, Reis FJJ, Arruda-Sanchez T, Caneiro JP, Meulders A (2025). Unraveling the role of fear and avoidance behavior in chronic musculoskeletal pain: from theory to physical therapy clinical practice. Braz J Phys Ther.

[11] Brea-Gómez B, Torres-Sánchez I, Ortiz-Rubio A (2021). Virtual reality in the treatment of adults with chronic low back pain: a systematic review and meta-analysis of randomized clinical trials. Int J Environ Res Public Health.

[12] Tack C (2021). Virtual reality and chronic low back pain. Disabil Rehabil Assist Technol.

[13] MacIntyre E, Sigerseth M, Larsen TF (2023). Get your head in the game: a replicated single-case experimental design evaluating the effect of a novel virtual reality intervention in people with chronic low back pain. J Pain.

[14] Chuan A, Zhou JJ, Hou RM, Stevens CJ, Bogdanovych A (2021). Virtual reality for acute and chronic pain management in adult patients: a narrative review. Anaesthesia.

[15] Krijn M, Emmelkamp PMG, Olafsson RP, Biemond R (2004). Virtual reality exposure therapy of anxiety disorders: a review. Clin Psychol Rev.

[16] Huang Q, Lin J, Han R, Peng C, Huang A (2022). Using virtual reality exposure therapy in pain management: a systematic review and meta-analysis of randomized controlled trials. Value Health.

[17] Malik K, Dua A (2026). Advancing Patient Care With Biofeedback.

[18] Lo HHM, Zhu M, Zou Z (2024). Immersive and nonimmersive virtual reality-assisted active training in chronic musculoskeletal pain: systematic review and meta-analysis. J Med Internet Res.

[19] Shchory S, Nitzan K, Harpaz G, Doron R (2024). Not just a game: the effect of active versus passive virtual reality experiences on anxiety and sadness. Virtual Real.

[20] Ahern MM, Dean LV, Stoddard CC (2020). The effectiveness of virtual reality in patients with spinal pain: a systematic review and meta-analysis. Pain Pract.

[21] Bordeleau M, Stamenkovic A, Tardif PA, Thomas J (2022). The use of virtual reality in back pain rehabilitation: a systematic review and meta-analysis. J Pain.

[22] Li R, Li Y, Kong Y (2024). Virtual reality-based training in chronic low back pain: systematic review and meta-analysis of randomized controlled trials. J Med Internet Res.

[23] Henríquez-Jurado JM, Osuna-Pérez MC, García-López H (2024). Virtual reality-based therapy for chronic low back and neck pain: a systematic review with meta-analysis. EFORT Open Rev.

[24] Kumar V, Vatkar AJ, Kataria M, Dhatt SS, Baburaj V (2024). Virtual reality is effective in the management of chronic low back ache in adults: a systematic review and meta-analysis of randomized controlled trials. Eur Spine J.

[25] Choi T, Heo S, Choi W, Lee S (2023). A systematic review and meta-analysis of the effectiveness of virtual reality-based rehabilitation therapy on reducing the degree of pain experienced by individuals with low back pain. Int J Environ Res Public Health.

[26] Page MJ, McKenzie JE, Bossuyt PM (2021). The PRISMA 2020 statement: an updated guideline for reporting systematic reviews. BMJ.

[27] Stone PW (2002). Popping the (PICO) question in research and evidence-based practice. Appl Nurs Res.

[28] Owen PJ, Miller CT, Mundell NL (2020). Which specific modes of exercise training are most effective for treating low back pain? Network meta-analysis. Br J Sports Med.

[29] Sterne JAC, Savović J, Page MJ (2019). RoB 2: a revised tool for assessing risk of bias in randomised trials. BMJ.

[30] Nikolakopoulou A, Higgins JPT, Papakonstantinou T (2020). CINeMA: an approach for assessing confidence in the results of a network meta-analysis. PLoS Med.

[31] Papakonstantinou T, Nikolakopoulou A, Higgins JPT, Egger M, Salanti G (2020). CINeMA: software for semiautomated assessment of the confidence in the results of network meta-analysis. Campbell Syst Rev.

[32] Salanti G, Del Giovane C, Chaimani A, Caldwell DM, Higgins JPT (2014). Evaluating the quality of evidence from a network meta-analysis. PLoS One.

[33] Balshem H, Helfand M, Schünemann HJ (2011). GRADE guidelines: 3. Rating the quality of evidence. J Clin Epidemiol.

[34] Liu Y, Béliveau A, Wei Y (2023). A gentle introduction to Bayesian network meta-analysis using an automated R package. Multivariate Behav Res.

[35] Barili F, Parolari A, Kappetein PA, Freemantle N (2018). Statistical Primer: heterogeneity, random- or fixed-effects model analyses?. Interact Cardiovasc Thorac Surg.

[36] Dias S, Welton NJ, Sutton AJ, Caldwell DM, Lu G, Ades AE (2014). NICE DSU Technical Support Document 4: Inconsistency in Networks of Evidence Based on Randomised Controlled Trials.

[37] Spiegelhalter DJ, Best NG, Carlin BP, Van Der Linde A (2002). Bayesian measures of model complexity and fit. J R Stat Soc Series B Stat Methodol.

[38] Röver C, Friede T (2023). Using the bayesmeta R package for Bayesian random-effects meta-regression. Comput Methods Programs Biomed.

[39] Rosenberger KJ, Duan R, Chen Y, Lin L (2021). Predictive P-score for treatment ranking in Bayesian network meta-analysis. BMC Med Res Methodol.

[40] Mbuagbaw L, Rochwerg B, Jaeschke R (2017). Approaches to interpreting and choosing the best treatments in network meta-analyses. Syst Rev.

[41] Afzal MW, Ahmad A, Mohseni Bandpei MA, Gilani SA, Hanif A, Waqas MS (2022). Effects of virtual reality exercises and routine physical therapy on pain intensity and functional disability in patients with chronic low back pain. J Pak Med Assoc.

[42] Čeko M, Baeuerle T, Webster L, Wager TD, Lumley MA (2024). The effects of virtual reality neuroscience-based therapy on clinical and neuroimaging outcomes in patients with chronic back pain: a randomized clinical trial. Pain.

[43] Eccleston C, Fisher E, Liikkanen S (2022). A prospective, double-blind, pilot, randomized, controlled trial of an “embodied” virtual reality intervention for adults with low back pain. Pain.

[44] Garcia LM, Birckhead BJ, Krishnamurthy P (2021). An 8-week self-administered at-home behavioral skills-based virtual reality program for chronic low back pain: double-blind, randomized, placebo-controlled trial conducted during COVID-19. J Med Internet Res.

[45] Hsieh RL, Chen YR, Lee WC (2025). Short-term effects of exergaming on patients with chronic low back pain: a single-blind randomized controlled trial. Musculoskelet Sci Pract.

[46] Kim SS, Min WK, Kim JH, Lee BH (2014). The effects of VR-based Wii Fit yoga on physical function in middle-aged female LBP patients. J Phys Ther Sci.

[47] Li Z, Yu Q, Luo H (2021). The effect of virtual reality training on anticipatory postural adjustments in patients with chronic nonspecific low back pain: a preliminary study. Neural Plast.

[48] Maddox T, Oldstone L, Sparks CY (2023). In-home virtual reality program for chronic lower back pain: a randomized sham-controlled effectiveness trial in a clinically severe and diverse sample. Mayo Clin Proc Digit Health.

[49] Massah N, Kahrizi S, Neblett R (2025). Comparison of the acute effects of virtual reality exergames and core stability exercises on cognitive factors, pain, and fear avoidance beliefs in people with chronic nonspecific low back pain. Games Health J.

[50] Matheve T, Bogaerts K, Timmermans A (2020). Virtual reality distraction induces hypoalgesia in patients with chronic low back pain: a randomized controlled trial. J Neuroeng Rehabil.

[51] McConnell R, Lane E, Webb G, LaPeze D, Grillo H, Fritz J (2024). A multicenter feasibility randomized controlled trial using a virtual reality application of pain neuroscience education for adults with chronic low back pain. Ann Med.

[52] Meinke A, Peters R, Knols RH, Swanenburg J, Karlen W (2022). Feedback on trunk movements from an electronic game to improve postural balance in people with nonspecific low back pain: pilot randomized controlled trial. JMIR Serious Games.

[53] Nambi G, Abdelbasset WK, Alrawaili SM, Alsubaie SF, Abodonya AM, Saleh AK (2021). Virtual reality or isokinetic training; its effect on pain, kinesiophobia and serum stress hormones in chronic low back pain: a randomized controlled trial. Technol Health Care.

[54] Nambi G, Abdelbasset WK, Elsayed SH (2020). Comparative effects of isokinetic training and virtual reality training on sports performances in university football players with chronic low back pain—randomized controlled study. Evid Based Complement Alternat Med.

[55] Sato T, Shimizu K, Shiko Y (2021). Effects of Nintendo Ring Fit Adventure exergame on pain and psychological factors in patients with chronic low back pain. Games Health J.

[56] Tuck N, Pollard C, Good C (2022). Active virtual reality for chronic primary pain: mixed methods randomized pilot study. JMIR Form Res.

[57] Yalfani A, Abedi M, Raeisi Z (2022). Effects of an 8-week virtual reality training program on pain, fall risk, and quality of life in elderly women with chronic low back pain: double-blind randomized clinical trial. Games Health J.

[58] Yilmaz Yelvar GD, Çırak Y, Dalkılınç M, Parlak Demir Y, Guner Z, Boydak A (2017). Is physiotherapy integrated virtual walking effective on pain, function, and kinesiophobia in patients with non-specific low-back pain? Randomised controlled trial. Eur Spine J.

[59] Zadro JR, Shirley D, Simic M (2019). Video-game-based exercises for older people with chronic low back pain: a randomized controlledtable trial (GAMEBACK). Phys Ther.

[60] Kim T, Lee J, Oh S, Kim S, Yoon B (2020). Effectiveness of simulated horseback riding for patients with chronic low back pain: a randomized controlled trial. J Sport Rehabil.

[61] Oh HW, Lee MG, Jang JY (2014). Time-effects of horse simulator exercise on psychophysiological responses in men with chronic low back pain. Isokinet Exerc Sci.

[62] Park JH, Lee SH, Ko DS (2013). The effects of the Nintendo Wii exercise program on chronic work-related low back pain in industrial workers. J Phys Ther Sci.

[63] Park S, Park S, Min S, Kim CJ, Jee YS (2020). A randomized controlled trial investigating the effects of equine simulator riding on low back pain, morphological changes, and trunk musculature in elderly women. Medicina (B Aires).

[64] Yoo JH, Kim SE, Lee MG (2014). The effect of horse simulator riding on visual analogue scale, body composition and trunk strength in the patients with chronic low back pain. Int J Clin Pract.

[65] Nambi G, Abdelbasset WK, Elsayed SH, Verma A, George JS, Saleh AK (2021). Clinical and physical efficiency of virtual reality games in soccer players with low back pain. Rev Bras Med Esporte.

[66] Dias S, Welton NJ, Caldwell DM, Ades AE (2010). Checking consistency in mixed treatment comparison meta-analysis. Stat Med.

[67] Vlaeyen JWS, Crombez G (2020). Behavioral conceptualization and treatment of chronic pain. Annu Rev Clin Psychol.

[68] Moustgaard H, Clayton GL, Jones HE (2020). Impact of blinding on estimated treatment effects in randomised clinical trials: meta-epidemiological study. BMJ.

[69] Chimenti RL, Post AA, Rio EK (2023). The effects of pain science education plus exercise on pain and function in chronic Achilles tendinopathy: a blinded, placebo-controlled, explanatory, randomized trial. Pain.

[70] Tatas Z, Kyriakou E, Koutsiouroumpa O, Seehra J, Mavridis D, Pandis N (2024). Most meta-analyses in oral health do not have conclusive and robust results. J Dent.

[71] Hayden JA, Ellis J, Ogilvie R, Malmivaara A, van Tulder MW (2021). Exercise therapy for chronic low back pain. Cochrane Database Syst Rev.

[72] Garofano M, Del Sorbo R, Calabrese M (2025). Remote rehabilitation and virtual reality interventions using motion sensors for chronic low back pain: a systematic review of biomechanical, pain, quality of life, and adherence outcomes. Technologies (Basel).

[73] Dias S, Welton NJ, Sutton AJ, Caldwell DM, Lu G, Ades AE (2013). Evidence synthesis for decision making 4: inconsistency in networks of evidence based on randomized controlled trials. Med Decis Making.

