# Supplementary material for: Efficacy of Various Virtual Reality Exposure Therapies for Chronic Low Back Pain: Systematic Review and Network Meta-Analysis
Source: J Med Internet Res. 2026 Jul 22;28:e90289. doi: 10.2196/90289 (PMC13392656; doi:10.2196/90289)
Supplement: Multimedia Appendix 1 [file jmir-v28-e90289-s001.doc]

**Appendix 1: Search strategies employed in each database.**

**(Last search on January 27, 2026)**

| **PubMed** | | **Total** |
| --- | --- | --- |
| **#1** | (((((((Virtual Reality[MeSH Terms]) OR (Virtual Reality[Title/Abstract])) OR (Reality, Virtual[Title/Abstract]) OR (Virtual Realit*, Educational[Title/Abstract])) OR (Educational Virtual Realit*[Title/Abstract])) OR (Realit*, Educational Virtual[Title/Abstract])) OR (Virtual Realit*, Instructional[Title/Abstract])) OR (Instructional Virtual Realit*[Title/Abstract])) OR (Realit*, Instructional Virtual[Title/Abstract]) | **27594** |
| **#2** | (((((Virtual Reality Exposure Therapy[MeSH Terms]) OR （Virtual Reality Exposure Therap*[Title/Abstract] )) OR (Virtual Reality Immersion Therap*[Title/Abstract])) OR (Virtual Reality Therap*[Title/Abstract])) OR (Reality Therap*, Virtual[Title/Abstract])) OR (Therap*, Virtual Reality[Title/Abstract]) | **1775** |
| **#3** | ((((((Exergaming[MeSH Terms]) OR (Exergam*[Title/Abstract])) OR (Active-Video Gaming*[Title/Abstract])) OR (Active Video Gaming[Title/Abstract])) OR (Gaming*, Active-Video[Title/Abstract])) OR (Virtual Reality Exercise*[Title/Abstract])) OR (Exercise*, Virtual Reality[Title/Abstract]) | **2030** |
| **#4** | (((((((((((((((((((Low Back Pain[MeSH Terms]) OR (Low Back Pain*[Title/Abstract])) OR (Back Pain*, Low[Title/Abstract])) OR (Pain*, Low Back[Title/Abstract])) OR (Lumbago[Title/Abstract])) OR (Lower Back Pain*[Title/Abstract])) OR (Back Pain*, Lower[Title/Abstract])) OR (Pain*, Lower Back[Title/Abstract])) OR (Low Back Ache*[Title/Abstract])) OR (Ache*, Low Back[Title/Abstract])) OR (Back Ache*, Low[Title/Abstract])) OR (Low Backache*[Title/Abstract])) OR (Backache*, Low[Title/Abstract])) OR (Low Back Pain, Postural[Title/Abstract])) OR (Postural Low Back Pain[Title/Abstract])) OR (Low Back Pain, Posterior Compartment[Title/Abstract])) OR (Low Back Pain, Recurrent[Title/Abstract])) OR (Recurrent Low Back Pain[Title/Abstract])) OR (Low Back Pain, Mechanical[Title/Abstract])) OR (Mechanical Low Back Pain[Title/Abstract]) | **53914** |
| **#5** | #1 OR #2 OR #3 | **29156** |
| **#6** | #4 AND #5 | **124** |

| **Web of Science** | | **Total** |
| --- | --- | --- |
| **#1** | TS=(Virtual Reality Exposure Therap* or Virtual Reality Immersion Therap* or Virtual Reality Therap* or Reality Therap*, Virtual or Therap*, Virtual Reality) | **14933** |
| **#2** | TS=(Virtual Reality or Reality, Virtual or Virtual Realit*, Educational or Educational Virtual Realit* or Realit*, Educational Virtual or Instructional Virtual Realit* or Realit*, Instructional Virtual or Virtual Realit*, Instructional) | **88860** |
| **#3** | TS=(Exergam* or Active-Video Gaming* or Active Video Gaming* or Gaming*, Active-Video or Virtual Reality Exercise* or Exercise*, Virtual Reality) | **7791** |
| **#4** | TS=(Low Back Pain* or Back Pain*, Low or Pain*, Low Back or Lumbago or Lower Back Pain* or Back Pain*, Lower or Pain*, Lower Back or Low Back Ache* or Ache*, Low Back or Back Ache*, Low or Low Backache* or Backache*, Low or Low Back Pain, Postural or Postural Low Back Pain or Low Back Pain, Posterior Compartment or Low Back Pain, Recurrent or Recurrent Low Back Pain or Low Back Pain, Mechanical or Mechanical Low Back Pain) | **104741** |
| **#5** | #1 OR #2 OR #3 | **91241** |
| **#6** | #4 AND #5 | **267** |

| **Embase** | | **Total** |
| --- | --- | --- |
| **#1** | 'virtual reality'/exp OR 'virtual reality' | **49938** |
| **#2** | 'virtual reality':ab,ti | **31949** |
| **#3** | 'exergaming'/exp OR 'exergaming' | **1552** |
| **#4** | 'active video gaming':ab,ti OR 'active videogaming':ab,ti OR 'exer-gaming':ab,ti OR 'virtual reality':ab,ti OR 'vr':ab,ti OR '-based exercise':ab,ti OR 'virtual reality exercise':ab,ti OR 'virtual reality-based exercise':ab,ti OR 'vr exercise':ab,ti OR 'vr exergaming':ab,ti OR 'vr-based exercise':ab,ti OR 'exergaming':ab,ti | **51686** |
| **#5** | 'virtual reality exposure therapy'/exp OR 'virtual reality exposure therapy' | **1536** |
| **#6** | 'virtual reality immersion therapy':ab,ti OR 'vr exposure therapy':ab,ti OR 'vr immersion therapy':ab,ti OR vret:ab,ti OR 'virtual reality exposure therapy':ab,ti | **531** |
| **#7** | 'low back pain'/exp OR 'low back pain' | **98575** |
| **#8** | 'acute low back pain':ab,ti OR 'back pain, low':ab,ti OR 'chronic low back pain':ab,ti OR 'loin pain':ab,ti OR 'low backache':ab,ti OR 'low backpain':ab,ti OR 'lowback pain':ab,ti OR 'lower back pain':ab,ti OR 'lumbago':ab,ti OR 'lumbal pain':ab,ti OR 'lumbal syndrome':ab,ti OR 'lumbalgesia':ab,ti OR 'lumbalgia':ab,ti OR 'lumbar pain':ab,ti OR 'lumbar spine syndrome':ab,ti OR 'lumbodynia':ab,ti OR 'lumbosacral pain':ab,ti OR 'lumbosacral root syndrome':ab,ti OR 'lumbosacroiliac strain':ab,ti OR 'pain, low back':ab,ti OR 'pain, lumbosacral':ab,ti OR 'strain, lumbosacroiliac':ab,ti OR 'low back pain':ab,ti | **68819** |
| **#9** | 'randomized controlled trial' | **1467470** |
| **#10** | #1 OR #2 OR #3 OR #4 OR #5 OR #6 | **69254** |
| **#11** | #7 OR #8 | **102449** |
| **#12** | #9 AND #10 AND#11 | **229** |

| **CINAHL** | | **Total** |
| --- | --- | --- |
| **#1** | MH "Virtual Reality" OR MH "Virtual Reality Exposure Therapy" OR MH "Augmented Reality" | **9209** |
| **#2** | TI (Virtual Reality Exposure Therapy or Virtual Reality Immersion Therapy or Virtual Reality Therap* or Reality Therap*, Virtual or Therap*, Virtual Reality or Virtual Reality or Reality, Virtual or Virtual Realit*, Educational or Educational Virtual Realit* or Realit*, Educational Virtual or Virtual Realit*, Instructional or Instructional Virtual Realit* or Realit*, Instructional Virtual or Active-Video Gaming* or Active Video Gaming or Gaming*, Active-Video or Virtual Reality Exercise* or Exercise*, Virtual Reality or Exergam*) OR AB (Virtual Reality Exposure Therapy or Virtual Reality Immersion Therapy or Virtual Reality Therap* or Reality Therap*, Virtual or Therap*, Virtual Reality or Virtual Reality or Reality, Virtual or Virtual Realit*, Educational or Educational Virtual Realit* or Realit*, Educational Virtual or Virtual Realit*, Instructional or Instructional Virtual Realit* or Realit*, Instructional Virtual or Active-Video Gaming* or Active Video Gaming or Gaming*, Active-Video or Virtual Reality Exercise* or Exercise*, Virtual Reality or Exergam*) | **9157** |
| **#3** | MH "Low Back Pain" OR MH "Back Pain" | **36893** |
| **#4** | TI (Low Back Pain* or Back Pain*, Low or Pain*, Low Back or Lumbago or Lower Back Pain* or Back Pain*, Lower or Pain*, Lower Back or Low Back Ache* or Ache*, Low Back or Back Ache*, Low or Low Backache* or Backache*, Low or Low Back Pain, Postural or Postural Low Back Pain or Low Back Pain, Posterior Compartment or Low Back Pain, Recurrent or Recurrent Low Back Pain or Low Back Pain, Mechanical or Mechanical Low Back Pain) OR AB (Low Back Pain* or Back Pain*, Low or Pain*, Low Back or Lumbago or Lower Back Pain* or Back Pain*, Lower or Pain*, Lower Back or Low Back Ache* or Ache*, Low Back or Back Ache*, Low or Low Backache* or Backache*, Low or Low Back Pain, Postural or Postural Low Back Pain or Low Back Pain, Posterior Compartment or Low Back Pain, Recurrent or Recurrent Low Back Pain or Low Back Pain, Mechanical or Mechanical Low Back Pain) | **25124** |
| **#5** | #1 OR #2 | **13808** |
| **#6** | #3 OR #4 | **43339** |
| **#7** | #5 AND #6 | **76** |

| **Scoups** | | **Total** |
| --- | --- | --- |
| **#1** | “Virtual Reality” OR “Virtual Reality, Education*” OR “Virtual Reality, Instruction*” | **198236** |
| **#2** | “Virtual Reality Exposure Therapy” OR “Virtual Reality Immersion Therapy” OR “Virtual Reality Therapy” | **2419** |
| **#3** | “Exergam*” OR “Active-Video Gaming” OR “Virtual Reality Exercise” | **3857** |
| **#4** | “Low* Back Pain” OR “Low Back Ache” OR “Low Back Pain, Mechanical” OR “Low Back Pain, Posterior Compartment” OR “Low Back Pain, Postural” OR “Low Back Pain, Recurrent” OR “Low Backache” OR “Lumbago” OR “Mechanical Low Back Pain” OR “Postural Low Back Pain” OR “Recurrent Low Back Pain” | **89013** |
| **#5** | #1 OR #2 OR #3 | **200918** |
| **#6** | #4 AND #5 | **208** |

| **Cochrane library** | | **Total** |
| --- | --- | --- |
| **#1** | MeSH descriptor: [Virtual Reality] explode all trees | **1692** |
| **#2** | (Reality*, Educational Virtual or Virtual Realit*, Educational or Instructional Virtual Realit* or Realit*, Instructional Virtual or Virtual Realit*, Instructional or Educational Virtual Realit* or Reality, Virtual):ti,ab,kw | **9599** |
| **#3** | MeSH descriptor: [Exergaming] explode all trees | **88** |
| **#4** | (Virtual Reality Exercise* or Exercise*, Virtual Reality or Active-Video Gaming* or Active Video Gaming* or Exergam* or Gaming*, Active-Video):ti,ab,kw | **2943** |
| **#5** | MeSH descriptor: [Virtual Reality Exposure Therapy] explode all trees | **437** |
| **#6** | (Reality Therap*, Virtual or Therapy*, Virtual Reality or Virtual Reality Immersion Therap* or Virtual Reality Therap*):ti,ab,kw | **3886** |
| **#7** | MeSH descriptor: [Low Back Pain] explode all trees | **6523** |
| **#8** | (Low Back Pain, Recurrent or Recurrent Low Back Pain or Mechanical Low Back Pain or Low Back Pain, Mechanical or Back Pain*, Low or Pain*, Lower Back or Low Backache* or Back Ache*, Low or Lower Back Pain* or Pain*, Low Back or Backache*, Low or Back Pain*, Lower or Lumbago or Low Back Pain* or Low Back Ache* or Ache*, Low Back or Low Back Pain, Posterior Compartment or Low Back Pain, Postural or Postural Low Back Pain):ti,ab,kw | **20245** |
| **#9** | #1 or #2 or #3 or #4 or #5 or #6 | **10601** |
| **#10** | #7 or #8 | **20245** |
| **#11** | #9 and #10 | **159** |
