# Supplementary material for: Efficacy of Various Virtual Reality Exposure Therapies for Chronic Low Back Pain: Systematic Review and Network Meta-Analysis
Source: J Med Internet Res. 2026 Jul 22;28:e90289. doi: 10.2196/90289 (PMC13392656; doi:10.2196/90289)
Supplement: Multimedia Appendix 2 [file jmir-v28-e90289-s002.doc]

Contents

[Figure S1. Pairwise Comparisons for Various Intervention Modalities of Virtual Reality Exposure Therapy.........................................................................................2](#__RefHeading___Toc201075612)

(A)Pain intensity;

(B) Function;

(C) Kinesiophobia.

[Figure S2. Rank heat plot of the different outcomes analyzed.................................3](#__RefHeading___Toc201075613)

[Figure S3. Funnel plot for publication bias.](#__RefHeading___Toc201075613)..............................................................4

(A)Pain intensity;

(B) Function;

(C) Kinesiophobia.

[Figure S4. Node-splitting Forest Plot of pain intensity............................................5](#__RefHeading___Toc201075619)

[Figure S5. Forest Plot for Heterogeneity Assessment..............................................6-8](#__RefHeading___Toc201075620)

[(A) Pain Intensity;](#__RefHeading___Toc201075620)

[(B) Function;](#__RefHeading___Toc201075620)

[(C) Kinesiophobia;](#__RefHeading___Toc201075620)

[Figure S6.Trace and Density Plots for Convergence Diagnosis...............................9-10](#__RefHeading___Toc201075621)

(A)Pain intensity;

(B) Function;

(C) Kinesiophobia.

[Text S1. Inclusion and exclusion criteria](#__RefHeading___Toc201075622)..................................................................11

[Table S1. Results of including studies.](#__RefHeading___Toc201075623)..................................................................12-19

Figure S1. Pairwise Comparisons for Various Intervention Modalities of Virtual Reality Exposure Therapy.

Grey cells indicate the exercise modality for each column.

Green cells indicate the comparisons are significan (confidence and credible intervals that don’t contain the 0).

1. Pain itensity


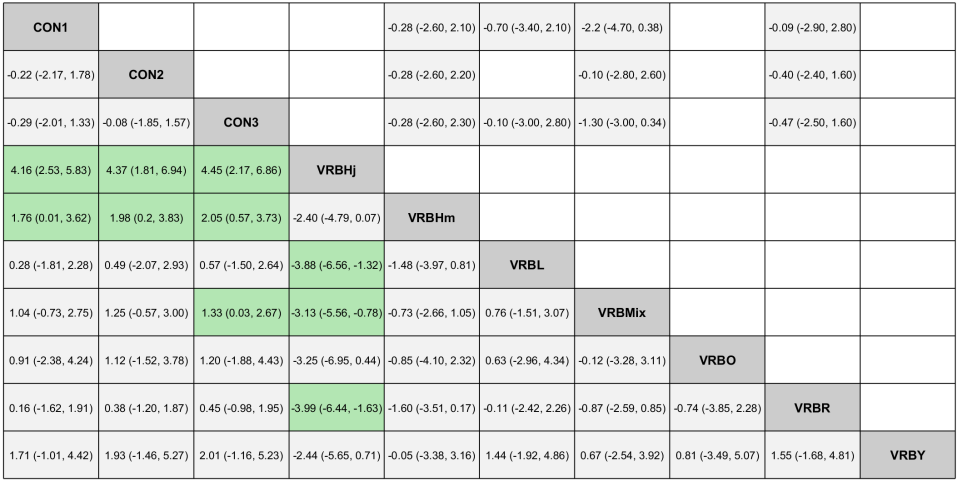


1. Function.


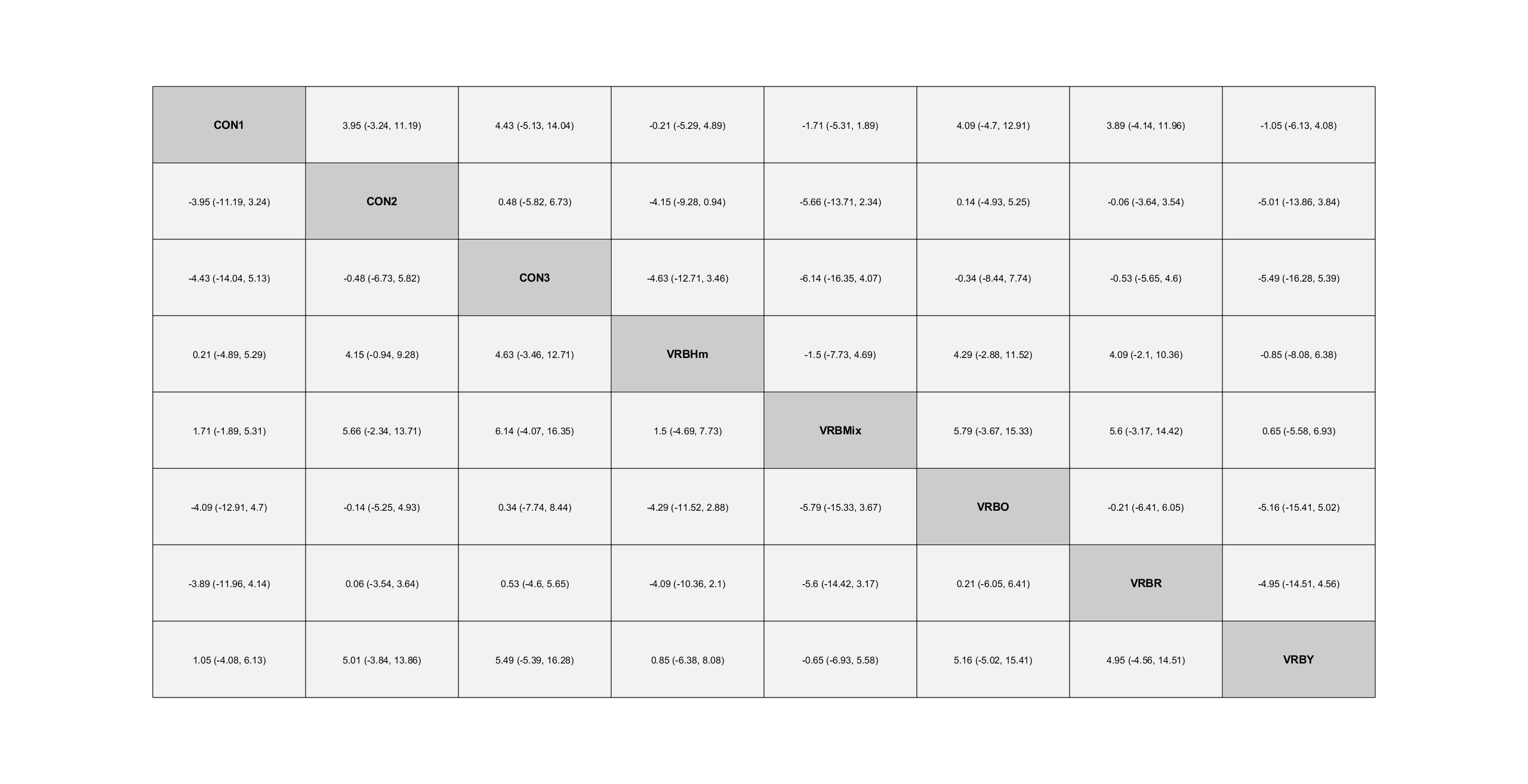


1. Kinesiophobia.


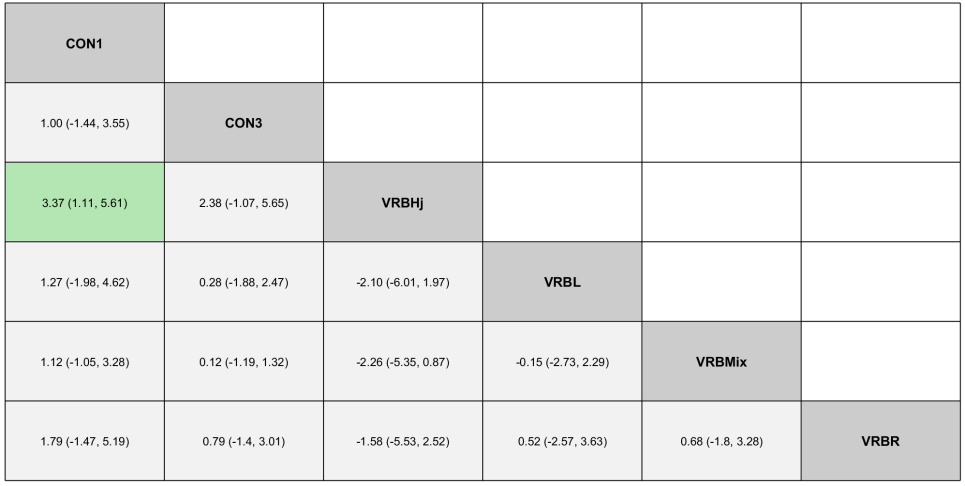


Figure S2. Rank heat plot of the different outcomes analyzed. Numbers and colors indicate the probability of being the first intervention recommended for a certain outcome.


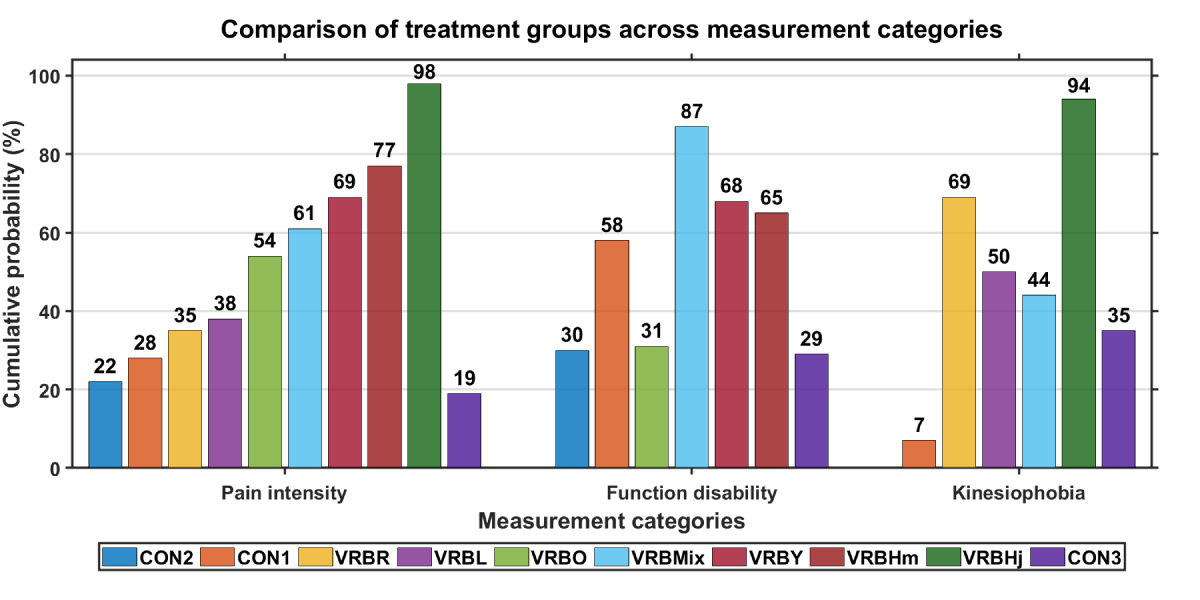


[Figure S3. Funnel plot for publication bias.](#__RefHeading___Toc201075613)


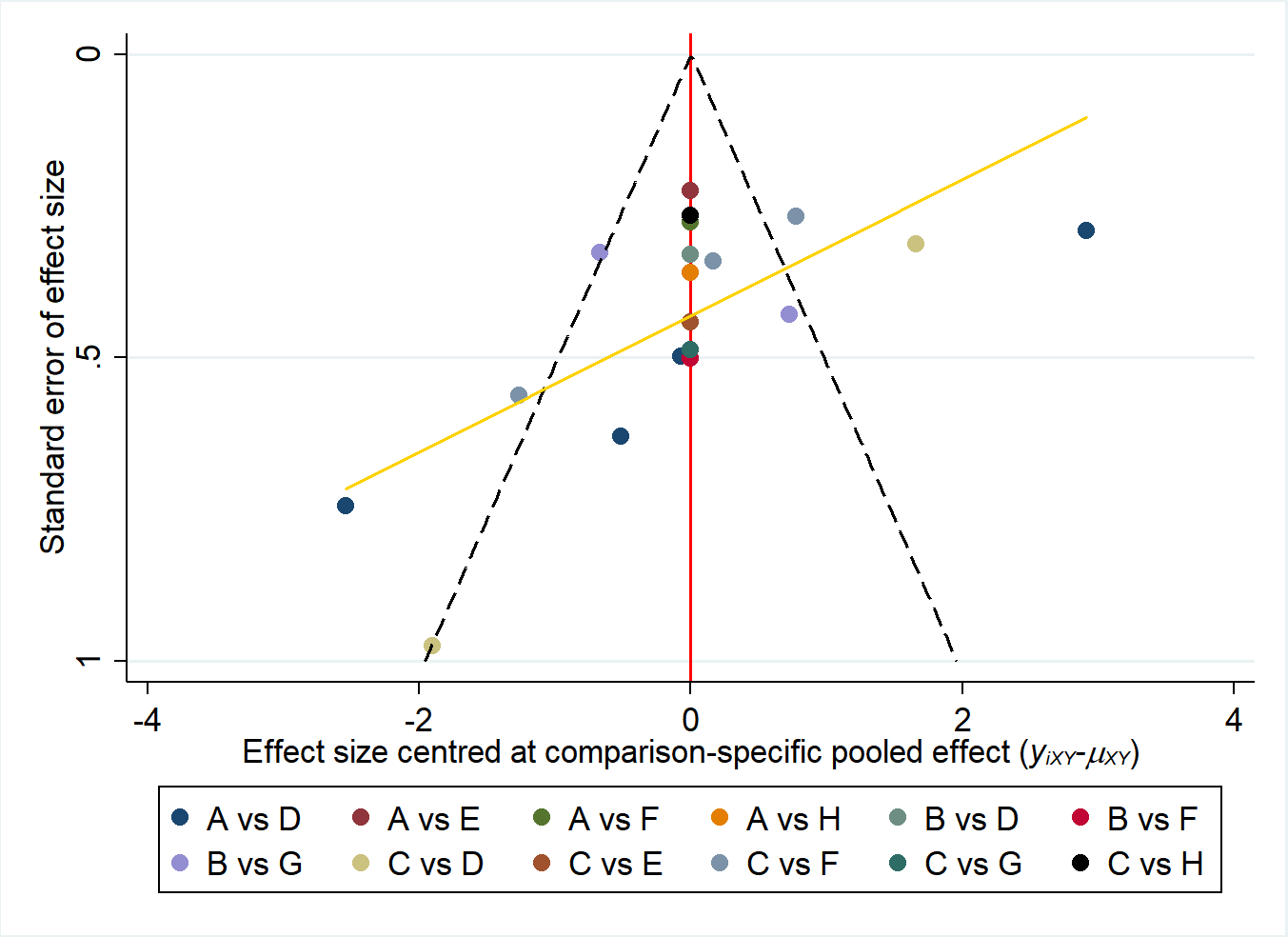
(A) Pain itensity.


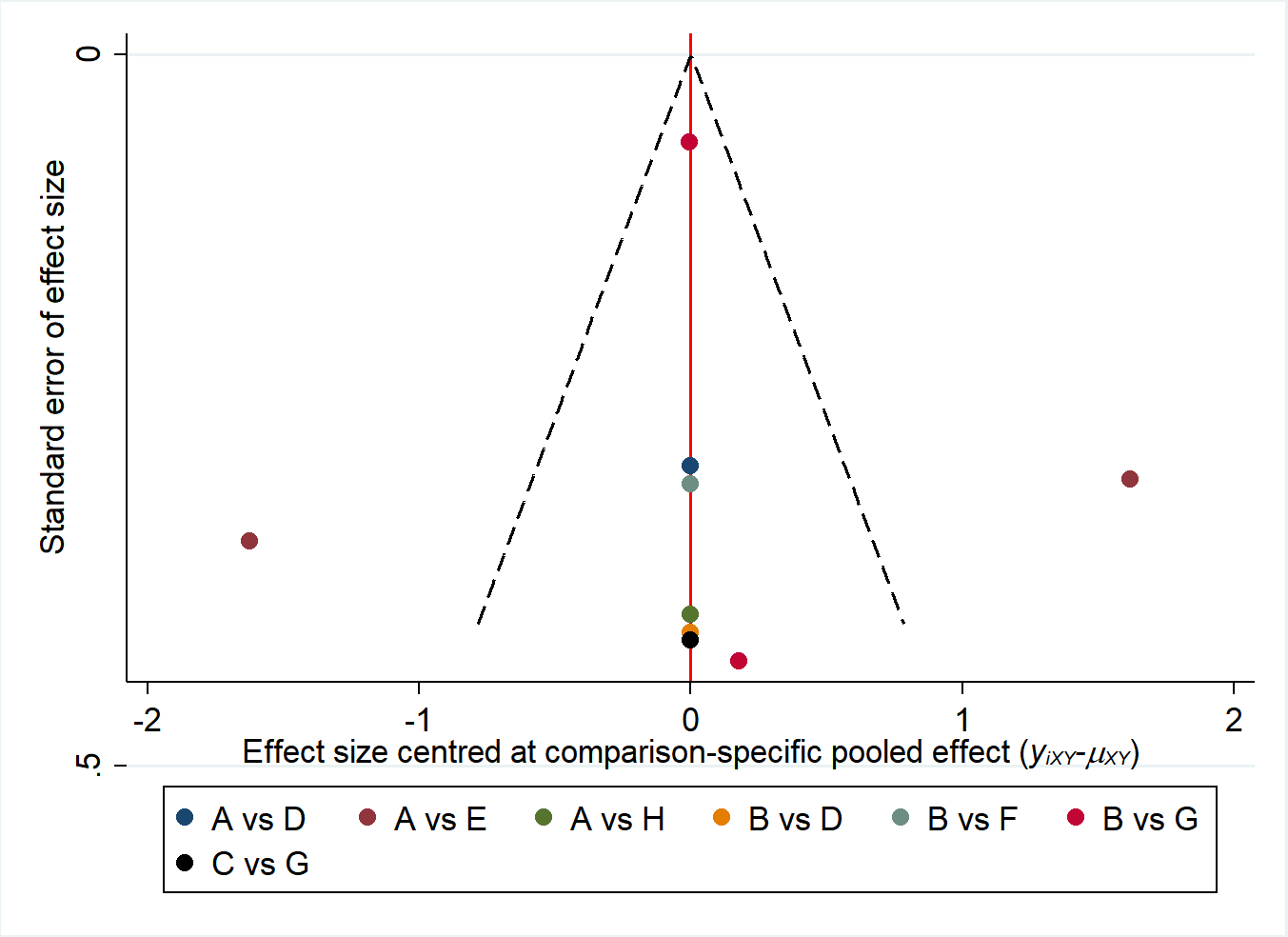
(B) Function.


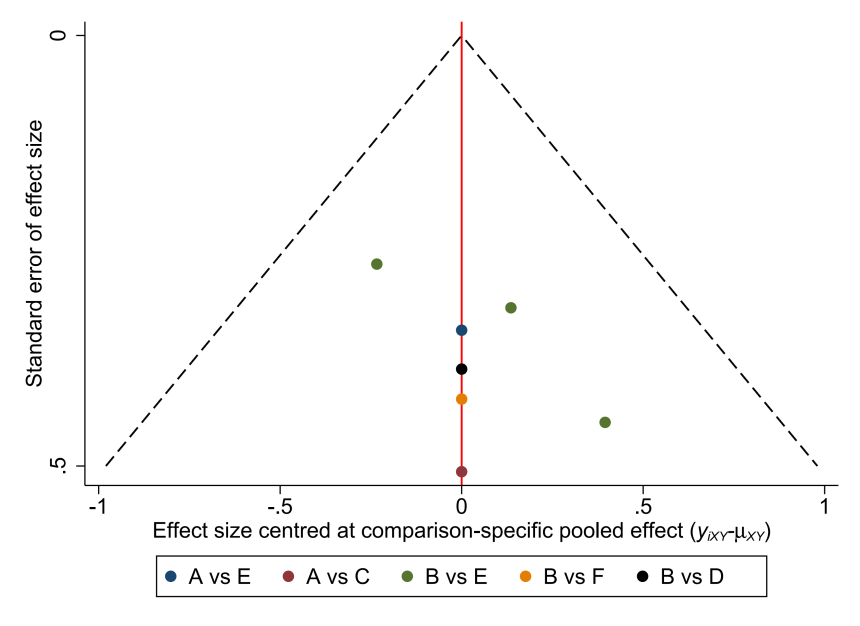
(C) Kinesiophobia.

Figure S4. Node-splitting Forest Plot of pain intensity.


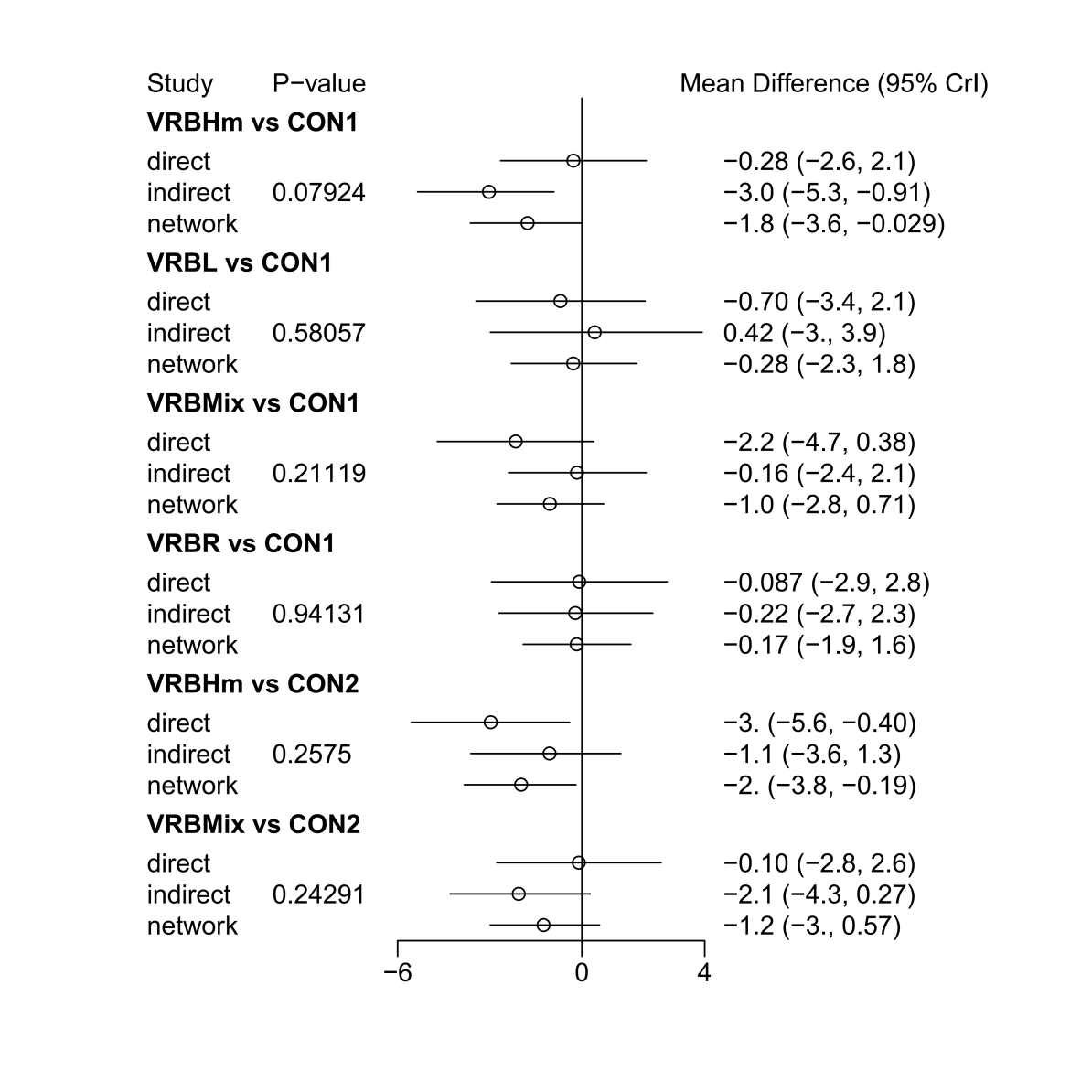

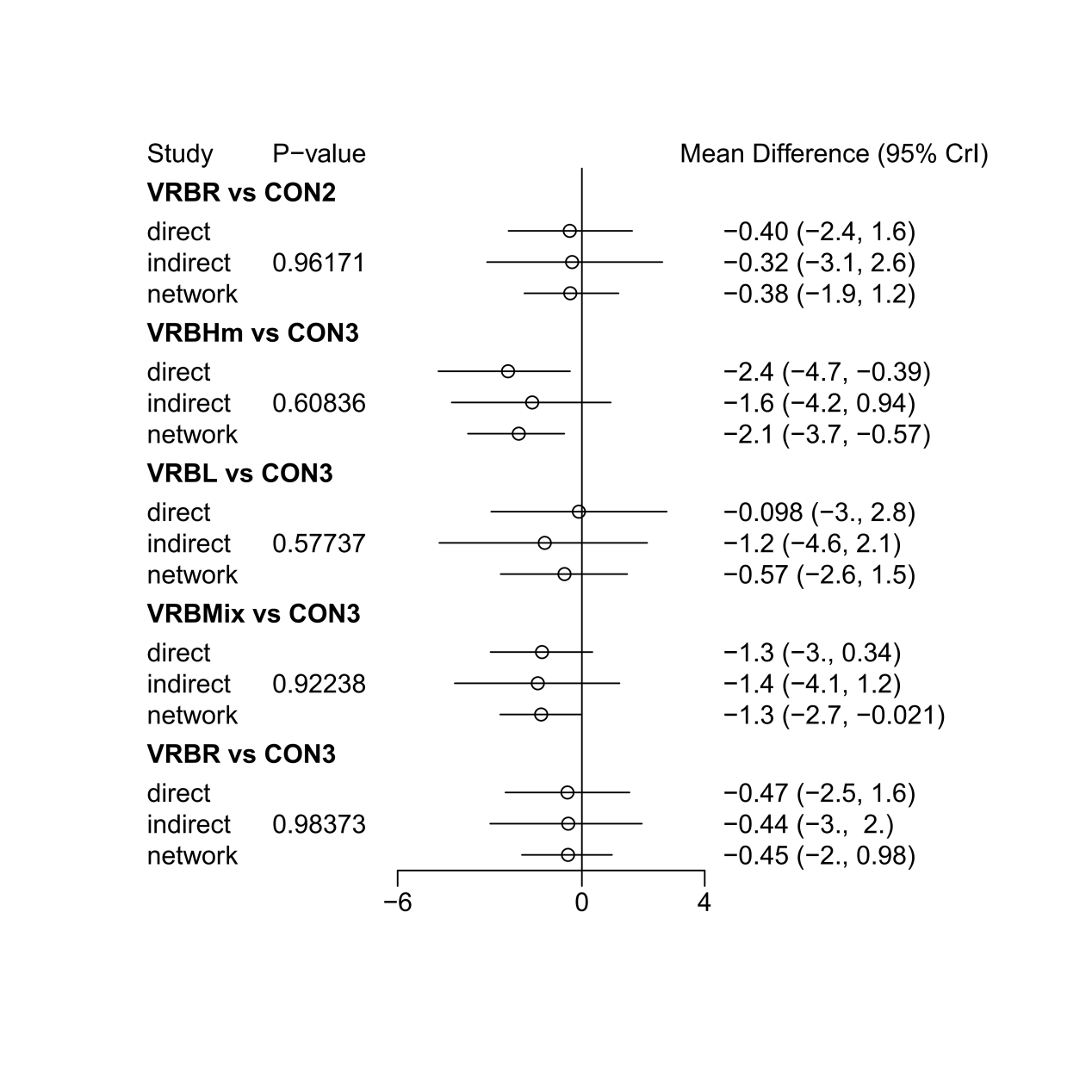


Figure S5. Forest Plot for Heterogeneity Assessment.

1. Pain itensity


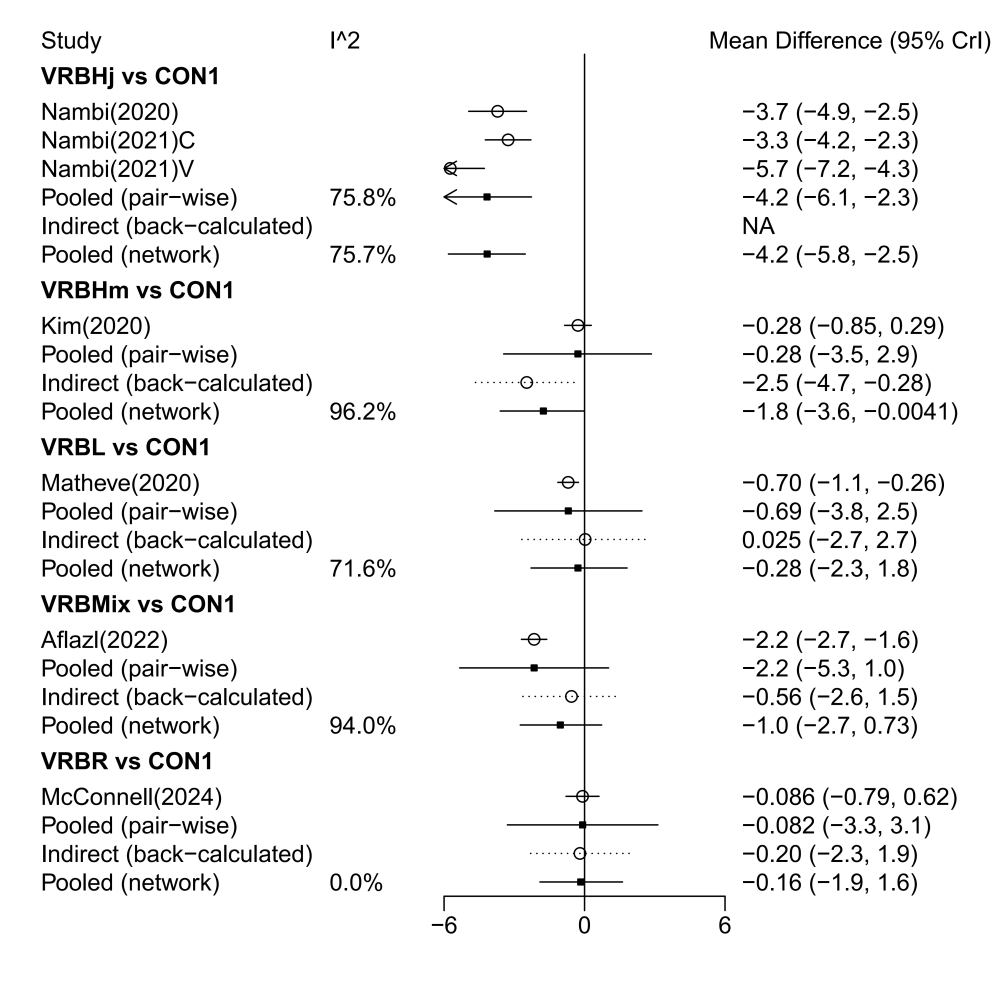

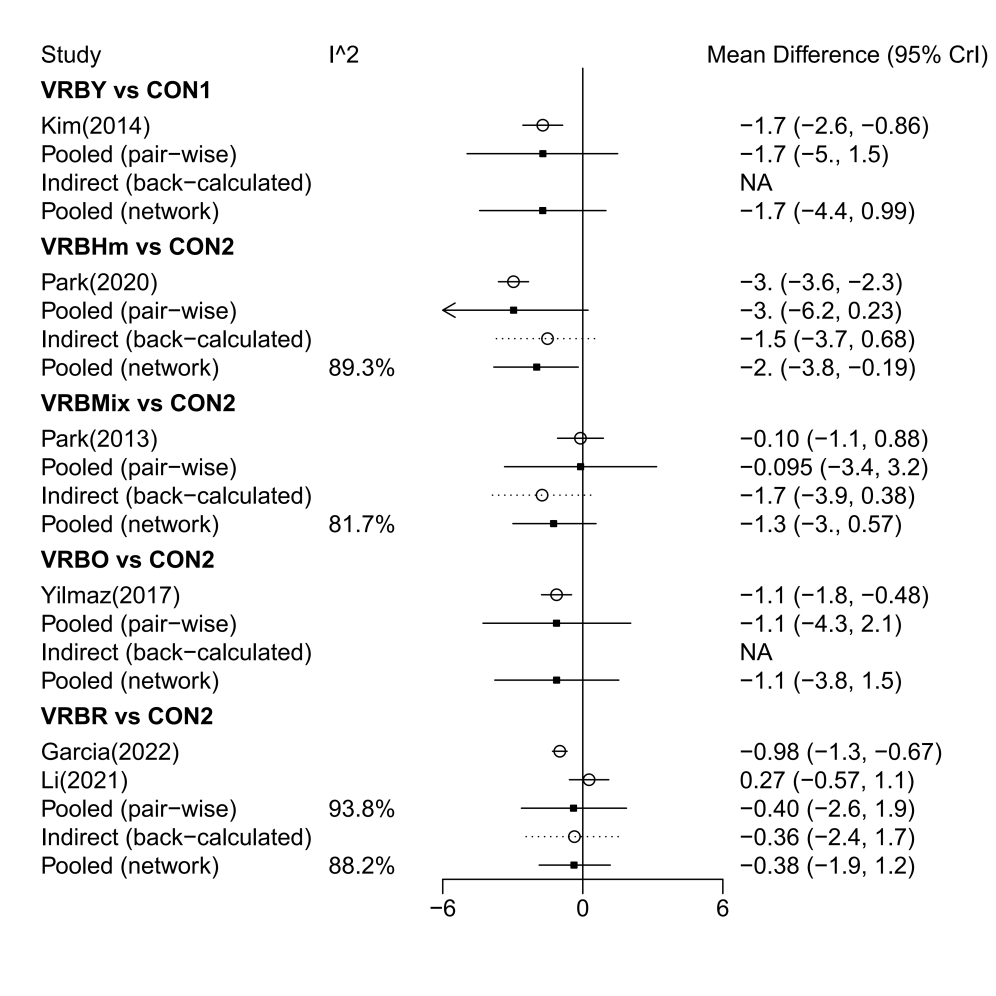

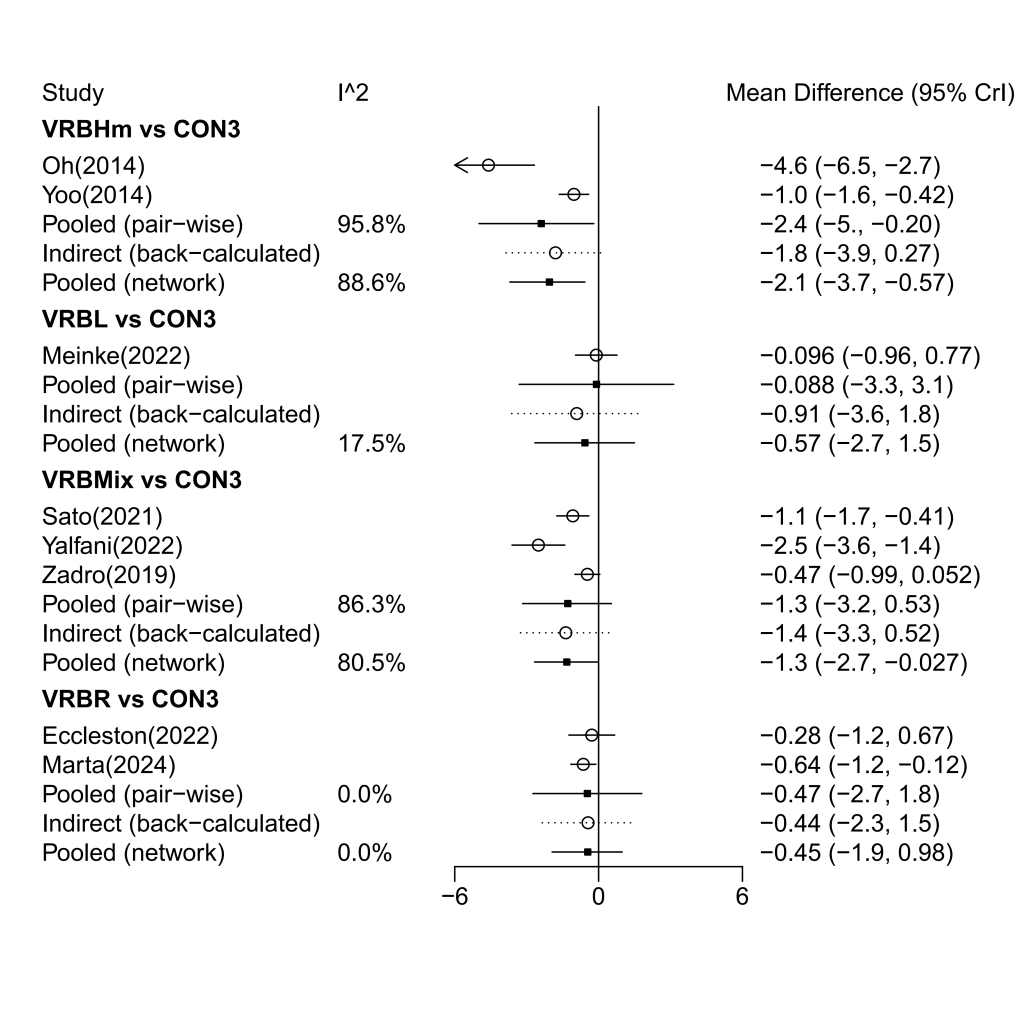


1. Function.


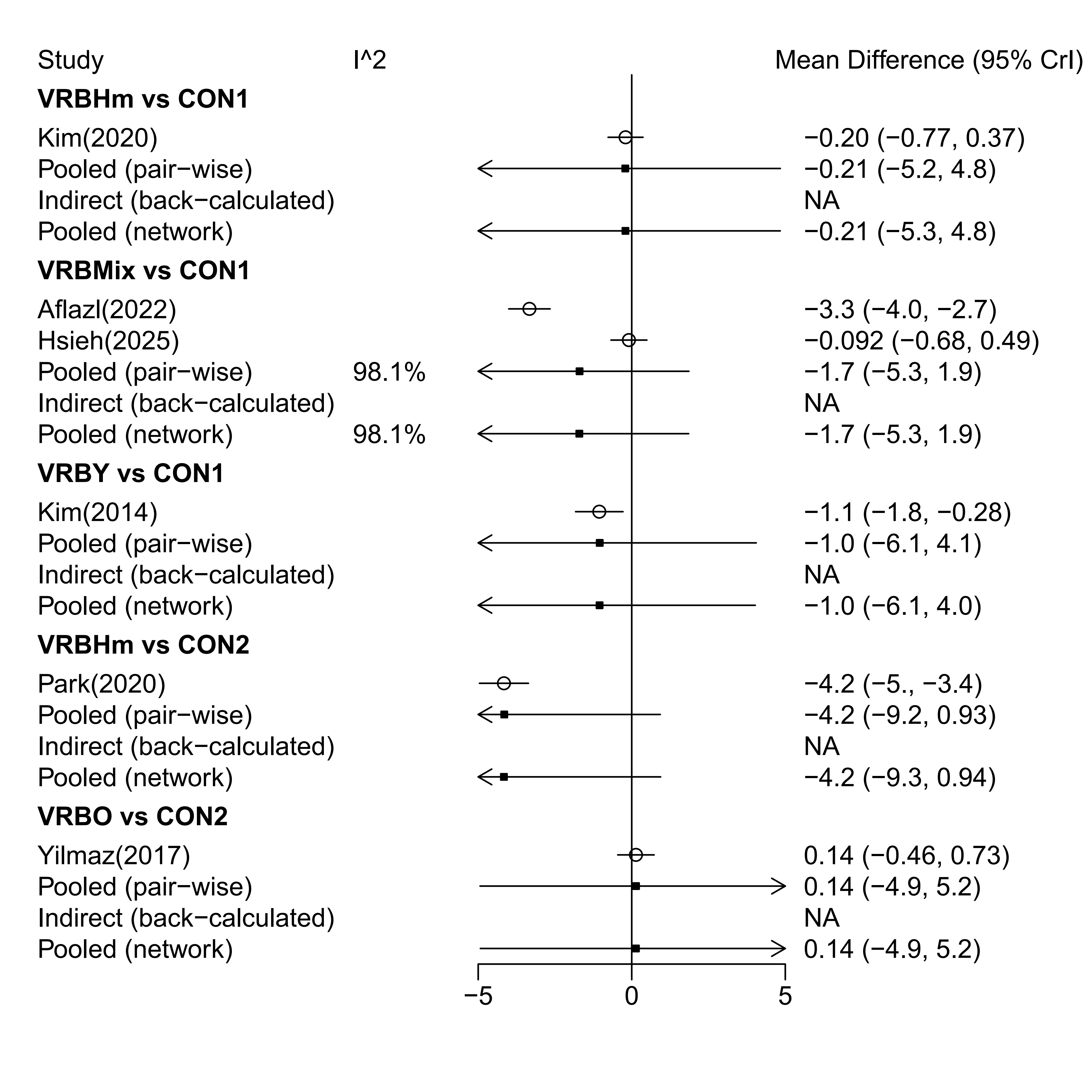

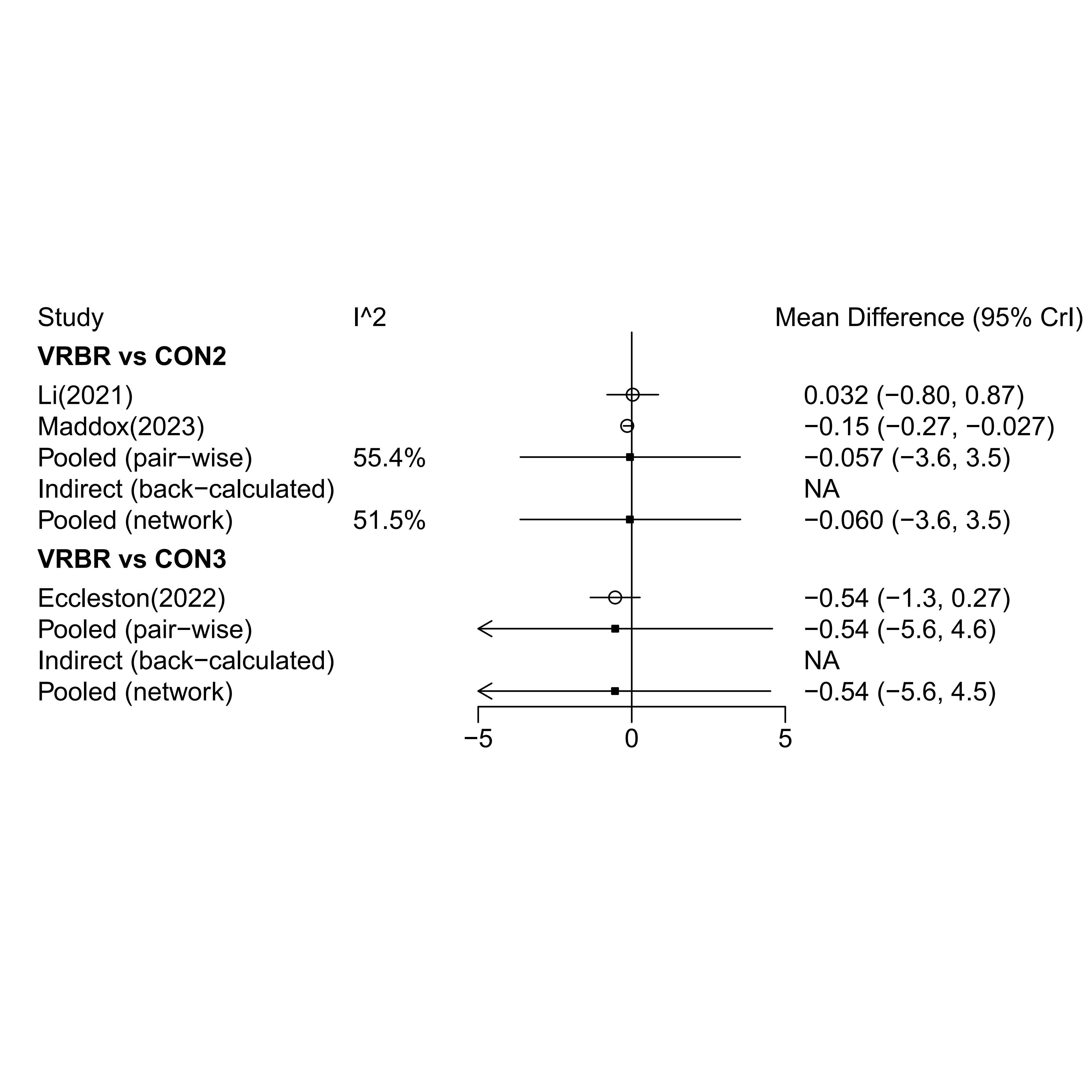


1. Kinesiophobia.


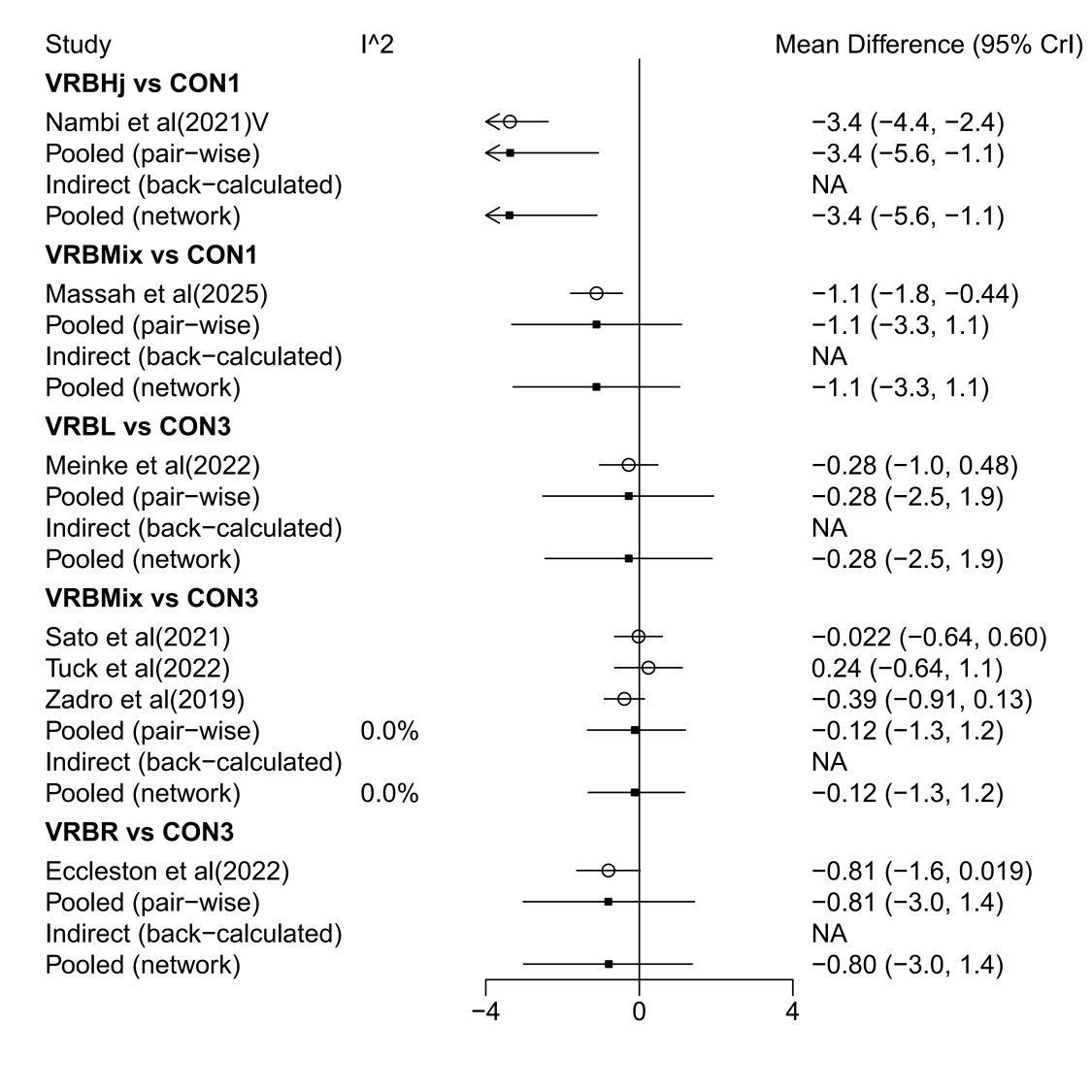


Figure S6. Trace and Density Plots for Convergence Diagnosis.


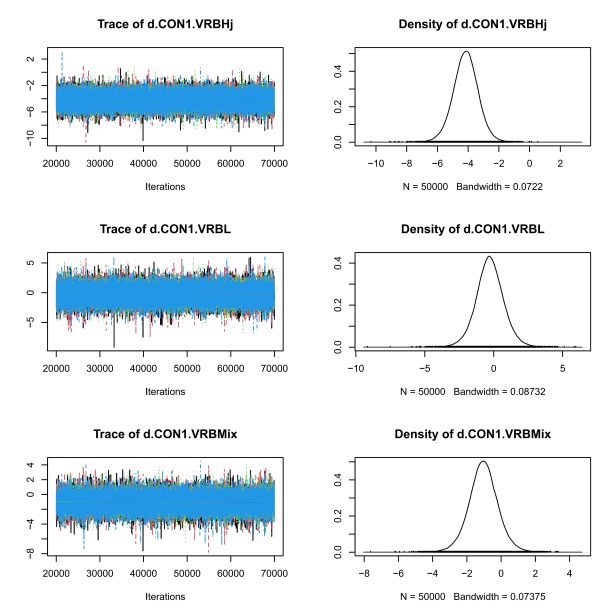

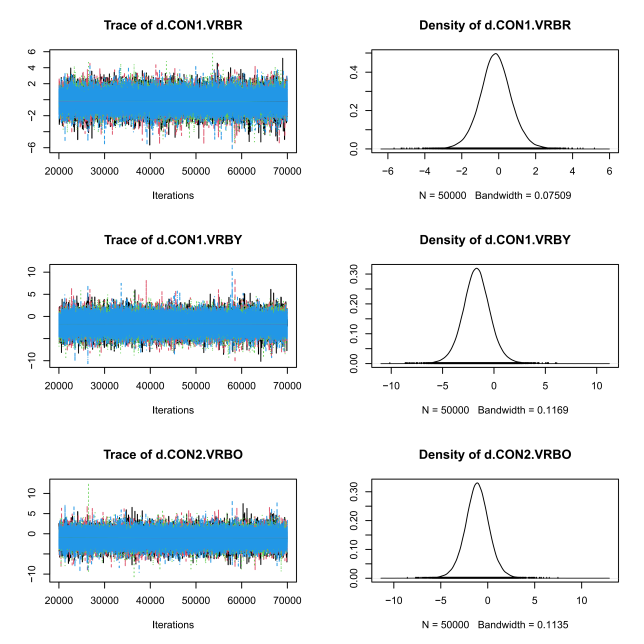

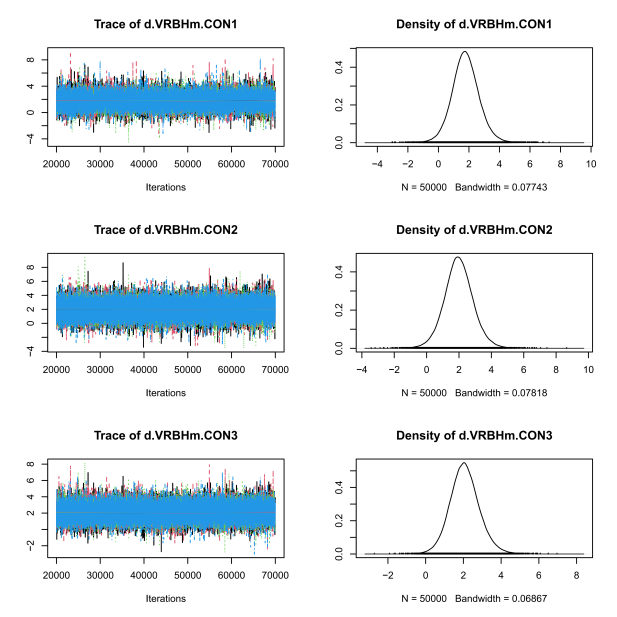

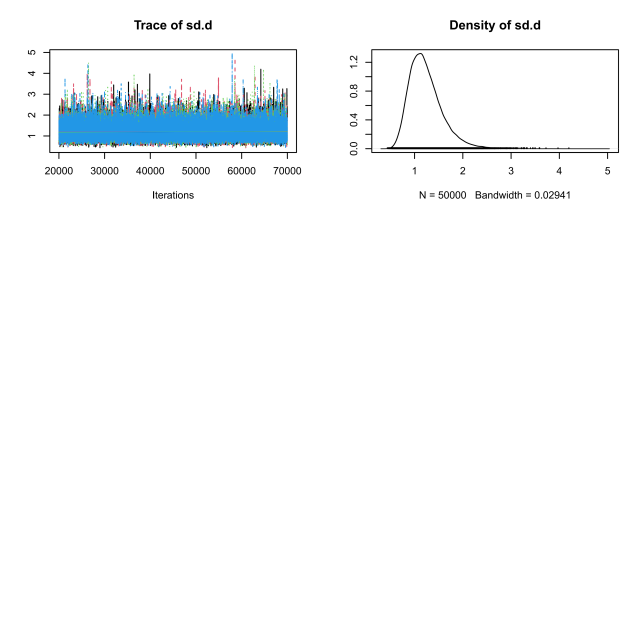


1. Pain itensity.


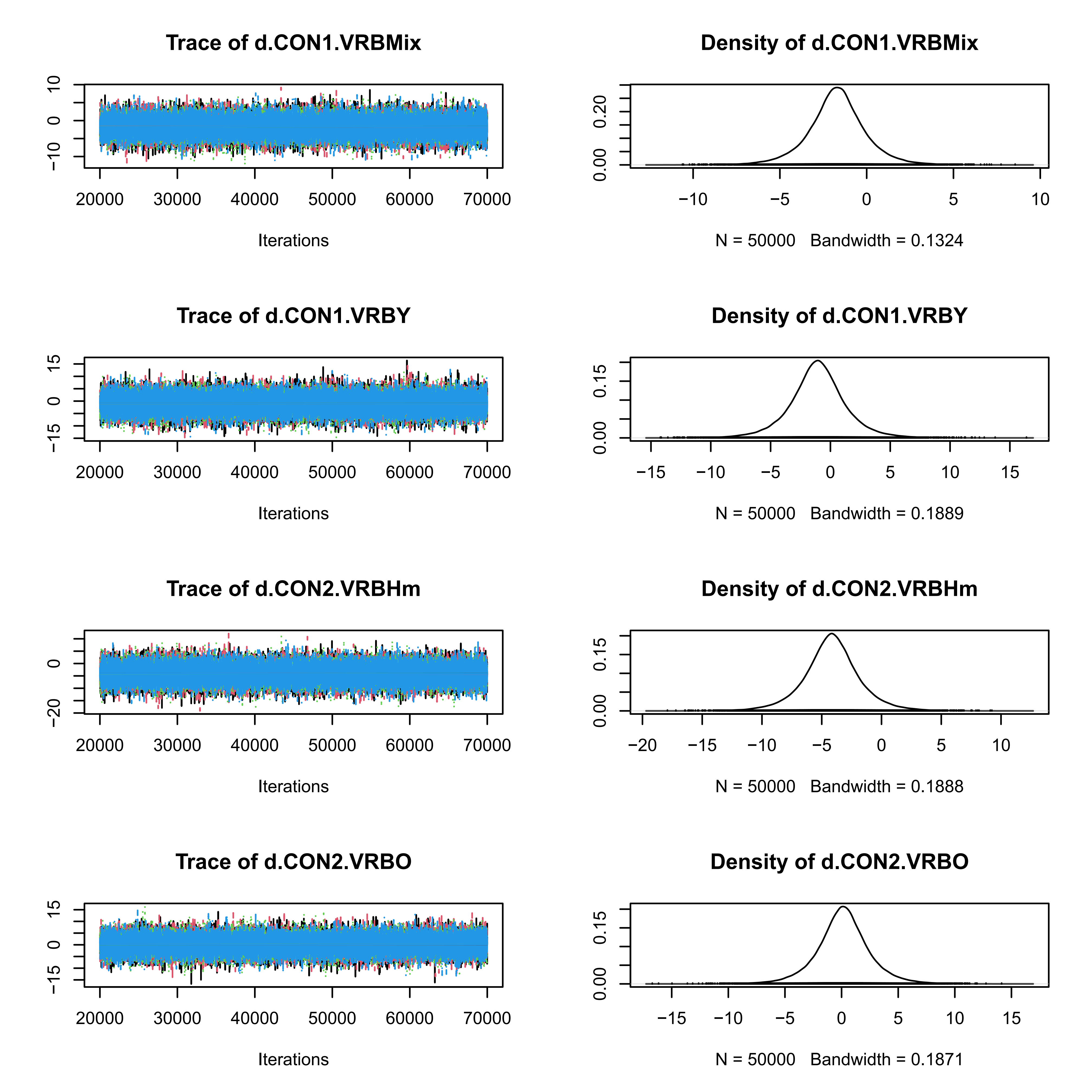

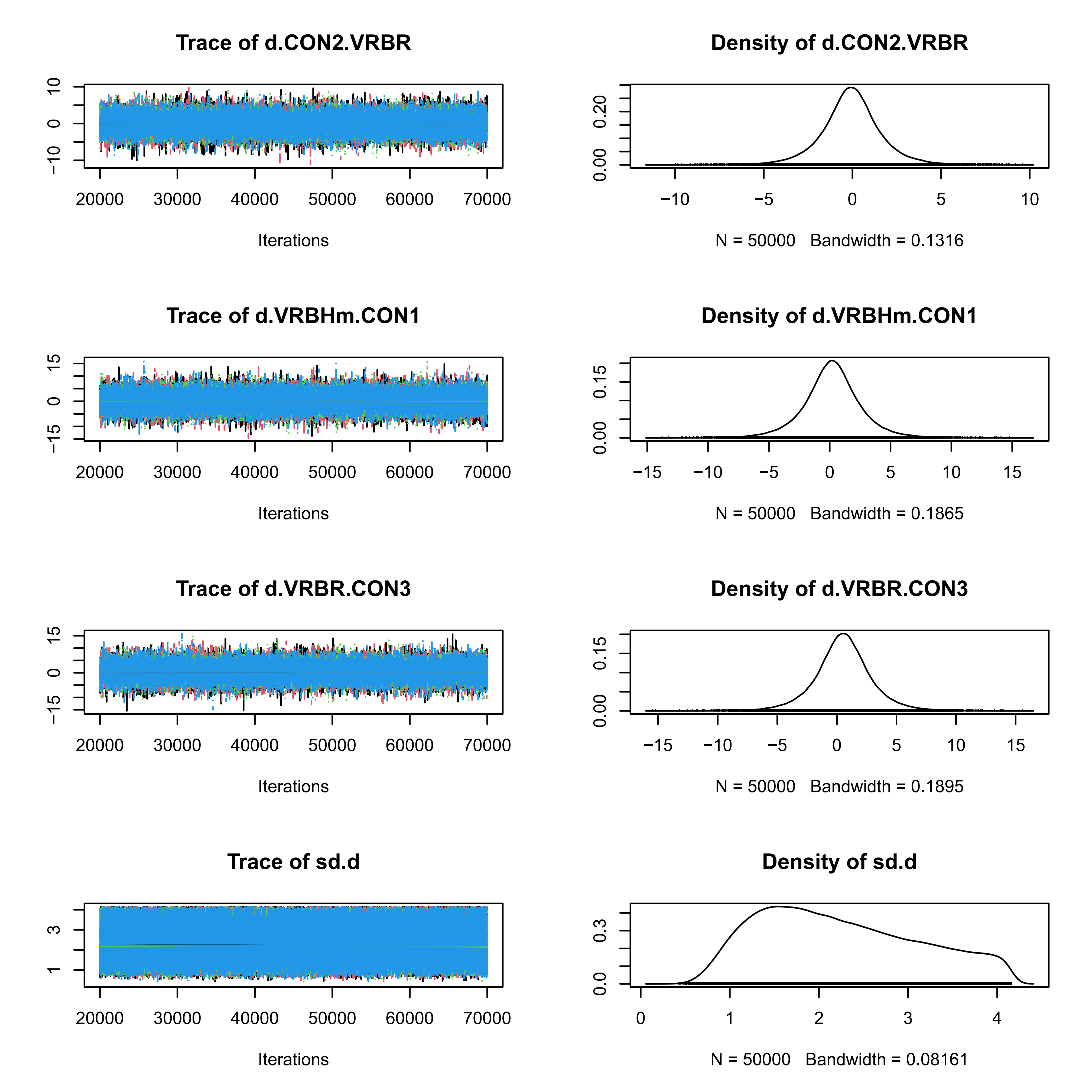


1. Function.


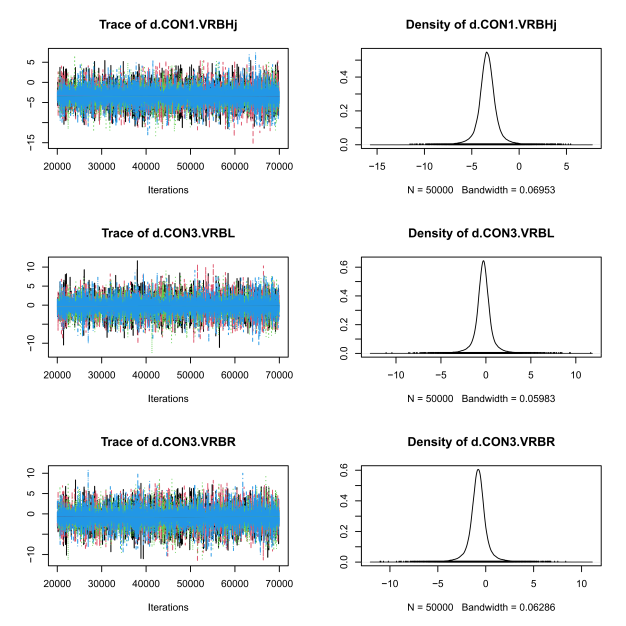

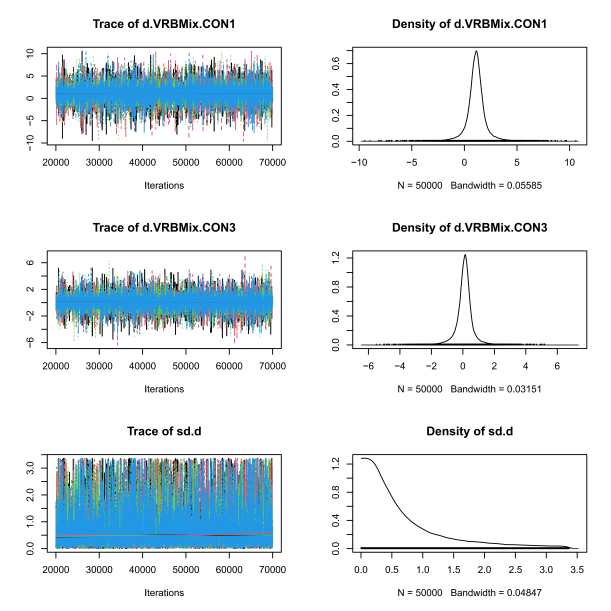


1. Kinesiophobia.

Text S1. Inclusion and exclusion criteria.

**Inclusion criteria**

• Paper type: randomized controlled trials of parallel groups

• Study subjects: participants with chronic low back pain (3 months or more)

• Interventions: virtual reality–based training as an intervention alone or in combination with physical therapy, use of virtual reality devices

• Outcomes: at least 1 of the following outcome measurements: pain (Visual Analog Scale, Numerical Rating Scale, and Defense and Veterans Pain Rating Scale), kinesiophobia (Tampa Scale of Kinesiophobia), or disability (Oswestry Disability Index)

• Language: written in English

**Exclusion criteria**

• Paper type: conference abstracts, reviews, and case reports

• Study subjects: specific low back pain or low back pain lasting for less than 3 months

• Interventions: no interventions involved virtual reality; The intervention consists of training using a VR device in conjunction with any other non-VR-based intervention (excluding physical agent therapy)

• Outcomes: without the required outcomes or unavailable data, and not enough information for analysis

• Language: non-English publications

Table S1. Results of including studies.

| **Study**  **(year)** | **Outcome measure** | **Time point** | **Statistical approach (within-group)** | **Effect size / Key result (within-group)** | **p value (within-group)** | **Statistical approach (between-group)** | **Effect size / Key result (between-group)** | **p value (between-group)** |
| --- | --- | --- | --- | --- | --- | --- | --- | --- |
| **Afzal et al (2022)**  **[41]** | Pain intensity (VAS) | 12 sessions (4 weeks) | Repeated measures ANOVA | EG: 6.62 ± 1.04 to 3.32 ± 0.81 | p<0.05 | Independent samples t-test | Difference in post-intervention means is 2.32. | p<0.05 |
|  |  |  | Repeated measures ANOVA | CG: 6.50 ± 1.24 to 1.00 ± 0.60 | p<0.05 |  |  |  |
|  | Functional disability (MODI) |  | Repeated measures ANOVA | EG: 65.08 ± 8.94 to 40.56 ± 8.59 | p<0.05 | Independent samples t-test | Difference in post-intervention means is 24.52. | p<0.05 |
|  |  |  | Repeated measures ANOVA | CG: 69.16 ± 9.13 to 16.04 ± 6.82 | p<0.05 |  |  |  |
| **Čeko et al. (2023)**  **[42]** | Pain intensity (BPI-SF) | Post-treatment (Week 8) | Mixed-effects model (Pre-Post change) | EG: 4.0 ± 1.2 to 2.5 ± 1.6 (Δ = -1.5) | NA | Mixed-effects model (Condition x Time interaction) | Hedge's g = 0.63 | p=0.014 |
|  |  |  | Mixed-effects model (Pre-Post change) | CG: 4.3 ± 1.4 to 3.8 ± 1.8 (Δ = -0.5) | NA |  |  |  |
|  | Pain interference / Functional disability (BPI-SF) |  | Mixed-effects model (Pre-Post change) | EG: 3.5 ± 1.4 to 1.5 ± 1.4 (Δ = -2.0) | NA | Mixed-effects model (Condition x Time interaction) | Hedge's g = 0.84 | p=0.002 |
|  |  |  | Mixed-effects model (Pre-Post change) | CG: 3.5 ± 1.6 to 2.8 ± 2.0 (Δ = -0.7) | NA |  |  |  |
|  | Kinesiophobia (TSK-11) |  | Mixed-effects model (Pre-Post change) | EG: 27.0 ± 4.9 to 21.3 ± 5.0 (Δ = -5.7) | NA | Mixed-effects model (Condition x Time interaction) | Hedge's g = 0.94 | p=0.007 |
|  |  |  | Mixed-effects model (Pre-Post change) | CG: 27.3 ± 4.9 to 25.6 ± 6.0 (Δ = -1.7) | NA |  |  |  |
| **Eccleston et al. (2022)**  **[43]** | Pain intensity (NRS average) | Post-treatment (6–8 weeks) | Paired comparison from baseline (Pre-Post change) | EG1: 5.7 ± 1.4 to 4.1 ± 1.7 (Δ = -1.6) | p=0.02 | Repeated-measures ANCOVA (Condition x Time interaction) | Estimate = -0.42 (SE 0.90), 95% CI: -2.25 to 1.41 | p=0.64 |
|  |  |  | Paired comparison from baseline (Pre-Post change) | CG: 5.4 ± 2.0 to 4.4 ± 2.4 (Δ = -1.0) | p=0.12 |  |  |  |
|  | Functional disability (ODI) |  | Paired comparison from baseline (Pre-Post change) | EG1: 34.8 ± 11.0 to 28.8 ± 15.6 (Δ = -6.0) | p=0.05 | Repeated-measures ANCOVA (Condition x Time interaction) | Estimate = -5.25 (SE 4.67), 95% CI: -14.77 to 4.27 | p=0.27 |
|  |  |  | Paired comparison from baseline (Pre-Post change) | CG: 33.8 ± 7.5 to 32.8 ± 8.6 (Δ = -1.0) | p=0.66 |  |  |  |
|  | Kinesiophobia (TSK) |  | Paired comparison from baseline (Pre-Post change) | EG1: 39.5 ± 4.4 to 33.7 ± 7.4 (Δ = -5.8) | p<0.001 | Repeated-measures ANCOVA (Condition x Time interaction) | Estimate = -6.16 (SE 2.35), 95% CI: -10.95 to -1.38 | p=0.01 |
|  |  |  | Paired comparison from baseline (Pre-Post change) | CG: 40.3 ± 4.6 to 39.8 ± 7.1 (Δ = -0.5) | p=0.84 |  |  |  |
| **Garcia et al. (2021)**  **[44]** | Pain intensity (DVPRS) | Day 56 (post-treatment) | Linear mixed model (time effect) | EG: d_rm = 1.31 (42.8% reduction) | p<0.001 | Linear mixed model (treatment group x time interaction) | Cohen's d = 0.49 (EG to CG) | p=0.001 (treatment effect);p<0.001 (treatment x time interaction) |
|  |  |  | Linear mixed model (time effect) | CG: d_rm = 0.75 (25.1% reduction) | NA |  |  |  |
| **Hsieh et al. (2025)**  **[45]** | Functional Disability (ODI) | T2 (2 weeks) | Paired t-test (improvement % vs baseline) | EG: 5.96% improvement | p=0.754 | Two-sample t-test (mean difference in improvement % between groups); Group × time interaction via RM-ANCOVA | Mean Difference (MD) = 9.11 (95% CI: 7.03, 11.19) | p<0.001 (for MD at T2);p=0.012 (Group × time interaction) |
|  |  |  | Paired t-test (improvement % vs baseline) | CG: -3.15% (worsening) | p=0.851 |  |  |  |
| **Kim et al (2014)**  **[46]** | Pain intensity (VAS) | 12 sessions (4 weeks) | Paired t-test | EG: 7.00±0.89 to 2.27±1.10 | p<0.01 | Repeated-measures ANCOVA | F=6.988 | p<0.05 |
|  |  |  | Paired t-test | CG: 6.95±0.79 to 4.63±1.91 | p<0.01 |  |  |  |
|  | Functional disability (ODI) |  | Paired t-test | EG: 34.91±6.19 to 13.82±7.65 | p<0.01 | Repeated-measures ANCOVA | F=4.510 | p<0.05 |
|  |  |  | Paired t-test | CG: 36.18±5.02 to 24.55±10.88 | p<0.01 |  |  |  |
| **Li et al (2021)**  **[47]** | Pain Intensity (VAS) | 2 weeks (10 sessions) | Two-way mixed-design repeated-measures ANOVA | EG：4.36 ± 1.36 to 3.18 ± 1.08 (Main effect of time: η²p=0.561) | p<0.001 | Two-way mixed-design repeated-measures ANOVA | Main effect of group(p=0.298,η²p=0.075) and time × group interaction(p=0.151,η²p=0.115). | p>0.05 |
|  |  |  | Two-way mixed-design repeated-measures ANOVA | CG：3.64 ± 1.36 to 2.18 ± 1.17 | NA |  |  |  |
|  | Functional Disability (ODI) |  | Two-way mixed-design repeated-measures ANOVA | EG: 15.65 ± 6.39 to 12.77 ± 6.28(Main effect of time: p=0.203). | p=0.203 | Two-way mixed-design repeated-measures ANOVA | Main effect of group (p=0.317,η²p=0.071) and time × group interaction (p=0.978,η²p=0.001). | p>0.05 |
|  |  |  | Two-way mixed-design repeated-measures ANOVA | CG: 12.72 ± 4.84 to 9.63 ± 7.20 | NA |  |  |  |
| **Maddox et al (2023)**  **[48]** | Functional Disability (ODI) | 8 weeks (Day 56) | Linear regression models (within-group change reported) | EG: 41.46 to 33.16 (-8.30 points, effect size=0.515) | p<0.001 | Linear regression models (between-group comparison) | 2.47 points, effect size=0.115. | p=0.004 |
|  |  |  | Linear regression models (within-group change reported) | CG: 40.95 to 35.12 (-5.83 points, effect size=0.34) | NA |  |  |  |
| **Massah et al (2025)**  **[49]** | Pain Intensity (VAS) | Single session (Acute) | Paired t-test / Wilcoxon signed-rank test | EG：4.25 ± 0.22 to 2.13, d=1.45 | p=0.000 | Independent t-test / Mann-Whitney U test | EG was significantly more effective than CG in reducing pain intensity (p=0.000). | p=0.000 |
|  |  |  | Paired t-test / Wilcoxon signed-rank test | CG：4.20 ± 0.31 to 3.68 | p=0.107 |  |  |  |
|  | Kinesiophobia (TSK) |  | Paired t-test / Wilcoxon signed-rank test | EG：43.5 ± 4.57 to 37.90 ± 4.09, d=2.18 | p=0.000 | Independent t-test / Mann-Whitney U test | EG was significantly more effective than CG in reducing TSK scores (p=0.000). | p=0.000 |
|  |  |  | Paired t-test / Wilcoxon signed-rank test | CG：No significant change. | NA |  |  |  |
| **Matheve et al (2020)**  **[50]** | Pain Intensity (NRS) | Single session (Acute) | Derived from Repeated Measures ANOVA | EG: 0.81 points. | NA | Repeated Measures ANOVA & Unpaired t-test | 1.31 points, d=0.85 (95% CI: 0.40-1.29). | p<0.003 |
|  |  |  | Derived from Repeated Measures ANOVA | CG: -0.50 points. | NA |  |  |  |
| **McConnell et al. (2024)**  **[51]** | Pain intensity (NPRS) | 6 weeks | Not explicitly reported | EG: 5.54 to 4.10 | NA | Independent samples t-test | Mean difference: 0.43 (95% CI: -1.09 to 1.95) | p=0.58 |
|  |  |  | Not explicitly reported | CG: 6.05 to 5.04 | NA |  |  |  |
|  | Functional disability (ODI) |  | Not explicitly reported | EG: 34.91 to 27.97 | NA | Independent samples t-test | Mean difference: 3.23 (95% CI: -5.31 to 11.77) | p=0.46 |
|  |  |  | Not explicitly reported | CG: 36.97 to 33.26 | NA |  |  |  |
| **Meinke et al. (2022)**  **[52]** | Pain intensity (NRS) | T2 to T3 (3 weeks) | Not explicitly reported | EG: -0.12 ± 1.12 | NA | Independent group t-tests or Wilcoxon rank-sum tests (ITT and PP) | Mean difference not directly reported; comparison based on change scores (control vs. intervention) | 0.28 (ITT) |
|  |  |  | Not explicitly reported | CG: 0.14 ± 1.18 | NA |  |  |  |
|  | Kinesiophobia (TSK-11) |  | Not explicitly reported | EG: -0.88 ± 3.18 | NA | Independent group t-test or Wilcoxon rank-sum test (ITT and PP) | Mean difference not directly reported; comparison based on change scores (control vs. intervention) | 0.23 (ITT) |
|  |  |  | Not explicitly reported | CG: -0.04 ± 2.63 | NA |  |  |  |
| **Nambi et al. (2020)**  **[54]** | Pain intensity (VAS) | 4 weeks | Repeated measures ANOVA (within each group) | EG: 7.1 ± 0.6 to 3.9 ± 0.5 | p<0.001 | One-way ANOVA (between groups at each time point) | NA | p<0.001 |
|  |  |  | Repeated measures ANOVA (within each group) | CG: 7.3 ± 0.6 to 6.2 ± 0.4 | p<0.001 |  |  |  |
| **Nambi et al. (2021)C**  **[65]** | Pain intensity (NPRS) | 4 weeks | Repeated measures ANOVA | EG1: 7.2 ± 0.6 to 4.1 ± 0.3 | p<0.001 | One-way ANOVA with post-hoc Benferroni test | NA | p<0.001 |
|  |  |  | Repeated measures ANOVA | CG: 7.4 ± 0.5 to 6.1 ± 0.5 | p<0.001 |  |  |  |
|  | Functional status (Quality of Life - Physical Fitness Index) |  | Repeated measures ANOVA | EG1: 8.59 ± 1.4 to 13.48 ± 1.3 | p<0.001 | One-way ANOVA with post-hoc Benferroni test | NA | p<0.001 |
|  |  |  | Repeated measures ANOVA | CG: 8.43 ± 1.4 to 9.98 ± 1.3 | p<0.001 |  |  |  |
| **Nambi et al. (2020)V**  **[53]** | Pain intensity (VAS) | 4 weeks | Repeated measures ANOVA | EG1: 7.5 ± 0.4 to 2.4 ± 0.2 | p<0.001 | One-way ANOVA with post-hoc Bonferroni | NA | p<0.001 |
|  |  |  | Repeated measures ANOVA | CG: 7.4 ± 0.4 to 4.5 ± 0.4 | p<0.001 |  |  |  |
|  | Kinesiophobia (TSK-17) |  | Repeated measures ANOVA | EG1: 57.52 ± 4.8 to 26.43 ± 3.5 | p<0.001 | One-way ANOVA with post-hoc Bonferroni | NA | p<0.001 |
|  |  |  | Repeated measures ANOVA | CG: 57.93 ± 4.3 to 46.21 ± 4.1 | p<0.001 |  |  |  |
| **Sato et al. (2021)**  **[55]** | Pain intensity - Low Back Pain (VAS) | 8 weeks | Paired t-test | EG: 7.42 ± 1.99 to 4.81 ± 3.01 (Δ = 2.61, 95% CI: 1.78 to 5.41) | p<0.01 | Unpaired t-test | Mean difference = -1.8 (95% CI: -4.28 to -0.17) | p=0.03 |
|  |  |  | Paired t-test | CG: No significant change reported | NA |  |  |  |
|  | Kinesiophobia (TSK) |  | Paired t-test | EG: 42.50 ± 5.94 to 39.69 ± 4.63 (Δ = 2.81, result not significant) | NA | Unpaired t-test | Mean difference = 3.47 (RFA 39.69 vs. Control 36.22) | p=0.45 |
|  |  |  | Paired t-test | CG: 38.92 ± 5.35 to 36.22 ± 3.23 | NA |  |  |  |
| **Tuck et al. (2022)**  **[56]** | Kinesiophobia (TSK-13) | 6 weeks | Not conducted (pilot study) | EG: Δ = -1.56 (SD 5.57) | NA | Hedges' g | g = 0.24 (95% CI: -0.63 to 1.10) | NA |
|  |  |  | Not conducted (pilot study) | CG: Δ = -2.90 (SD 5.17) | NA |  |  |  |
| **Yalfani et al. (2022)**  **[57]** | Pain intensity (VAS) | 8 weeks | Not explicitly reported | EG: 6.73 ± 2.42 to 2.19 ± 1.49 | NA | One-way ANCOVA | F = 117.002, ES = 0.84 | p=0.001 |
|  |  |  | Not explicitly reported | CG: 6.79 ± 1.99 to 7.54 ± 1.9 | NA |  |  |  |
| **Yilmaz Yelvar et al. (2017)**  **[58]** | Pain intensity (VAS) | 2 weeks (after 10 sessions) | Repeated-measures ANCOVA (time effect) | EG: 6.00 ± 1.06 to 2.52 ± 1.80 | p<0.001 | Repeated-measures ANCOVA (group × time interaction) | F(1,40) = 6.28, p = 0.02, Cohen's d = 0.28 | p=0.02 (interaction) |
|  |  |  | Repeated-measures ANCOVA (time effect) | CG: 5.63 ± 2.36 to 4.90 ± 3.39 | p<0.001 |  |  |  |
|  | Functional disability (ODI) |  | Repeated-measures ANCOVA (time effect) | EG: 20.72 ± 7.22 to 16.90 ± 5.45 | p<0.001 | Repeated-measures ANCOVA (group × time interaction) | F(1,38) = 0.08, p = 0.78, Cohen's d = 0.47 | p=0.78 (interaction) |
|  |  |  | Repeated-measures ANCOVA (time effect) | CG: 26.10 ± 11.03 to 21.05 ± 9.94 | p<0.001 |  |  |  |
|  | Kinesiophobia (TKS) |  | Repeated-measures ANCOVA (time effect) | EG: 43.72 ± 4.32 to 29.56 ± 4.04 | p<0.001 | Repeated-measures ANCOVA (group × time interaction) | F(1,38) = 0.32, p = 0.57, Cohen's d = 0.82 | p=0.57 (interaction); post-treatment between-group p=0.023 |
|  |  |  | Repeated-measures ANCOVA (time effect) | CG: 40.36 ± 5.61 to 38.70 ± 5.44 | p<0.001 |  |  |  |
| **Zadro et al. (2019)**  **[59]** | Pain intensity (NRS) | 8 weeks (post-intervention) | Paired comparison from baseline | EG: 5.2 ± 1.6 to 3.8 ± 2.4 | NA | Linear regression (adjusted for baseline and function) | β = −1.07, 95% CI: −2.11 to −0.03 | p=0.04 |
|  |  |  | Paired comparison from baseline | CG: 4.8 ± 1.7 to 4.4 ± 2.3 | NA |  |  |  |
|  | Kinesiophobia (TSK) |  | Paired comparison from baseline | EG: 33.6 ± 6.1 to 32.3 ± 7.1 | NA | Linear regression (adjusted for baseline and function) | β = −2.97, 95% CI: −6.14 to 0.21 | p=0.07 |
|  |  |  | Paired comparison from baseline | CG: 34.7 ± 5.8 to 35.9 ± 5.8 | NA |  |  |  |
| **Kim et al. (2020)**  **[60]** | Pain intensity (NRS) | 6 months (Follow-up) | Two-way repeated measures ANOVA (intragroup effect over time) | EG: 4.70 to 1.42 (ES: -0.24) | p<0.05 | Two-way repeated measures ANOVA (interaction effect: Group x Time) | F = 1.696 | p = 0.211 |
|  |  |  | Two-way repeated measures ANOVA (intragroup effect over time) | CG: 4.73 to 1.22 (ES: -0.40) | p<0.05 |  |  |  |
|  | Functional Disability (ODI) |  | Two-way repeated measures ANOVA (intragroup effect over time) | EG: 20.24 to 8.28 (ES: -0.24) | p<0.05 | Two-way repeated measures ANOVA (interaction effect: Group x Time) | F = 1.848 | p = 0.201 |
|  |  |  | Two-way repeated measures ANOVA (intragroup effect over time) | CG: 21.77 to 9.23 (ES: -0.41) | p<0.05 |  |  |  |
| **Oh et al. (2014)**  **[61]** | Pain Intensity (VAS - Back Pain) | 8 weeks (Post-intervention) | Pre-Post comparison within each group (data reported as Δ% change) | EG: -59.44% | NA | Kruskal-Wallis rank test on delta values (Δ%) across all four groups, followed by post-hoc Tukey ranks test | Z = 6.634 | Overall: p = 0.001 |
|  |  |  | Pre-Post comparison within each group (data reported as Δ% change) | CG: +16.17% | NA |  |  |  |
| **Park et al. (2013)**  **[62]** | Pain Intensity (VAS) | 8 weeks (Post-intervention) | Paired t-test (Pre-Post comparison within each group) | EG: 6.75 ± 1.38 to 5.87 ± 1.12 | p<0.05 | One-way ANOVA (for group differences in pre-test values). Specific between-group post-test comparisons not reported. | NA | NA |
|  |  |  | Paired t-test (Pre-Post comparison within each group) | CG: 6.62 ± 0.74 to 4.87 ± 0.83 | p<0.05 |  |  |  |
| **Park et al. (2020)**  **[63]** | Pain intensity (VAS) | 12 weeks | Δ% change from baseline | EG: Δ% = –71.45% reduction | NA | ANCOVA | F = 184.428, significant group × time interaction | p<0.001 |
|  |  |  | Δ% change from baseline | CG: Δ% = +5.06% increase | NA |  |  |  |
|  | Functional disability (ODI) |  | Δ% change from baseline | EG: Δ% = –59.79% reduction | NA | ANCOVA | F = 370.083, significant group × time interaction | p<0.001 |
|  |  |  | Δ% change from baseline | CG: Δ% = +3.93% increase | NA |  |  |  |
| **Yoo et al. (2014)**  **[64]** | Pain intensity (VAS) | 8 weeks | Wilcoxon matched-pairs signed-rank test | EG: Δ% = –41.77% | p=0.001 | Mann-Whitney U test (on Δ% values) | Z = –2.536 | p=0.011 |
|  |  |  | Wilcoxon matched-pairs signed-rank test | CG: Δ% = –25.00% | p=0.002 |  |  |  |
